# Molecular Breeding Strategy and Challenges Towards Improvement of Downy Mildew Resistance in Cauliflower (*Brassica oleracea* var. *botrytis* L.)

**DOI:** 10.3389/fpls.2021.667757

**Published:** 2021-07-20

**Authors:** Ranjan K. Shaw, Yusen Shen, Zhenqing Zhao, Xiaoguang Sheng, Jiansheng Wang, Huifang Yu, Honghui Gu

**Affiliations:** Institute of Vegetables, Zhejiang Academy of Agricultural Sciences, Hangzhou, China

**Keywords:** cauliflower, downy mildew, molecular breeding, QTL mapping, resistance gene

## Abstract

Cauliflower (*Brassica oleracea* var. *botrytis* L.) is one of the important, nutritious and healthy vegetable crops grown and consumed worldwide. But its production is constrained by several destructive fungal diseases and most importantly, downy mildew leading to severe yield and quality losses. For sustainable cauliflower production, developing resistant varieties/hybrids with durable resistance against broad-spectrum of pathogens is the best strategy for a long term and reliable solution. Identification of novel resistant resources, knowledge of the genetics of resistance, mapping and cloning of resistance QTLs and identification of candidate genes would facilitate molecular breeding for disease resistance in cauliflower. Advent of next-generation sequencing technologies (NGS) and publishing of draft genome sequence of cauliflower has opened the flood gate for new possibilities to develop enormous amount of genomic resources leading to mapping and cloning of resistance QTLs. In cauliflower, several molecular breeding approaches such as QTL mapping, marker-assisted backcrossing, gene pyramiding have been carried out to develop new resistant cultivars. Marker-assisted selection (MAS) would be beneficial in improving the precision in the selection of improved cultivars against multiple pathogens. This comprehensive review emphasizes the fascinating recent advances made in the application of molecular breeding approach for resistance against an important pathogen; Downy Mildew (*Hyaloperonospora parasitica*) affecting cauliflower and *Brassica oleracea* crops and highlights the QTLs identified imparting resistance against this pathogen. We have also emphasized the critical research areas as future perspectives to bridge the gap between availability of genomic resources and its utility in identifying resistance genes/QTLs to breed downy mildew resistant cultivars. Additionally, we have also discussed the challenges and the way forward to realize the full potential of molecular breeding for downy mildew resistance by integrating marker technology with conventional breeding in the post-genomics era. All this information will undoubtedly provide new insights to the researchers in formulating future breeding strategies in cauliflower to develop durable resistant cultivars against the major pathogens in general and downy mildew in particular.

## Introduction

Cauliflower (*Brassica oleracea* var. *botrytis* L.) is one of the most important vegetables of *Brassica oleracea* grown and consumed worldwide. Cauliflower belongs to the family *Brassicaceae* and is one of the three diploid species of *Brassica* in the triangle of U (Nagaharu, 1935). It contains ‘CC’ genome and has nine pairs of chromosomes (2n = 2x = 18). *Brassica oleracea* and its C-genome wild relatives were diversified in the northeast Mediterranean region (Arias et al., 2014), brought to the east Mediterranean region and eventually spread throughout Europe and became fully domesticated. World’s total production of cauliflower and broccoli was 2.65 million tones with a yield of 186,937 hg/ha and the total harvested area was 1.4 million hectares in 2018 (FAOSTAT, 2018). Due to its nutrient rich quality and medicinal value, production of cauliflower is increasing in each passing year throughout the world. But its production is severely constrained by biotic stresses and is prone to attack by a range of fungal, bacterial and viral diseases causing considerable damages at different phenological stages of the crop. Among several pathogens, downy mildew is one of the most harmful and devastating disease posing a serious threat to cauliflower productivity since many years. The disease attack reduces the quantity and quality of the produce imposing a great limitation in realizing the yield potential of the crop. So, an effective strategy is required for better management of downy mildew disease in cauliflower. Cultural, chemical and biological control of down mildew may not be very effective, economical and durable. ‘Host-plant resistance’ is widely recognized as the least expensive, easiest, safest and the most effective method of disease control (Agrios, 2005). Breeding for disease resistance helps to assemble desirable combinations of resistance genes in the new or existing varieties. But conventional plant breeding takes considerable time (5–10 years) to develop resistant varieties making the process expensive, time intensive and requires artificial screening facilities to grow the pathogens. In this scenario, molecular markers offer an opportunity to overcome the problems associated with the conventional breeding methodologies by reducing the reliance on laborious large-scale screening procedures. Molecular marker technology is integrated into the existing plant breeding programs (called molecular breeding) allowing the researchers to access, transfer and combine disease resistance genes faster and precisely which was otherwise not possible previously. Molecular breeding approach helps in early generation detection of resistance alleles at any prevailing environment well before the trait is expressed phenotypically imparting high confidence in selection. The selected genotypes can be used for hybridization in the same season speeding up the varietal development for disease resistance by a factor of 2–3 times. If disease resistance is governed by recessive genes, MAS allows the breeders to identify heterozygous plants carrying a recessive resistance allele which is difficult to detect phenotypically. MAS offers potential savings when there is a need to select for multiple resistance genes simultaneously whereas in conventional methods, it is often necessary to conduct separate trials to screen for each disease. Since years, classical breeders have developed many disease resistant varieties; however, the time-consuming process of making crosses, backcrosses and the selection of the desired resistant phenotypes makes it difficult to react adequately to the evolution of new virulent pathogens making these varieties ineffective to the new virulent strains. As cauliflower is attacked by a wide range of diseases, demand for developing multiple disease-resistant varieties is growing. The evolution of new virulent races of pathogens requires a persistent and continuous effort in disease management. Molecular breeding offers rapid and targeted selection in enhancing varietal development for disease resistance. So, the focus has shifted towards molecular breeding which could facilitates the combination of multiple resistant genes in the elite parental background of cauliflower.

**FIGURE 1:**
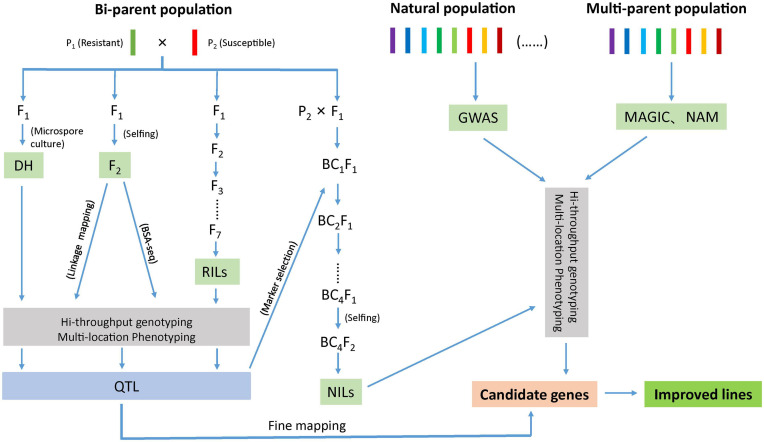
Schematic diagram representing different approaches to develop improved lines for disease resistance.

Identification of markers in close proximity with the desired trait can be accomplished through bi-parental QTL mapping using pedigree-based populations or by association mapping approach using natural population (Figure 1). In bi-parental population, QTL mapping is restricted to loci segregating between the two parents (Buckler and Thornsberry, 2002) where as in association mapping, the marker–trait association is established as a result of non-random segregation between the alleles. Another approach, nested association mapping population (NAM) holds the promise of combining the advantages of two methods (bi-parental linkage mapping and association mapping) in identifying quantitative loci (Yu and Buckler, 2006). Recently, a multi-parent advanced generation intercross (MAGIC) population strategy has gained momentum as it helps in interrogating multiple alleles with better mapping resolution (Cavanagh et al., 2008). Another strategy; ‘QTL-seq’ rapidly identifies the QTLs compared to conventional QTL analysis as this approach uses next-generation sequencing technologies to carry out whole genome resequencing of two DNA bulks of progeny (20–50 individuals) from a segregating population showing contrasting phenotypes (Takagi et al., 2013; Figure 1).

In this review, we are going to summarize the latest advancements and progresses made in cauliflower and *Brassica oleracea* crops against downy mildew disease by molecular breeding approach. We will also discuss the challenges, future strategy and the way forward to breed resistant cultivars supported by genomic approaches that can be implemented in the future for developing downy mildew resistant cultivars. The information and knowledge gained from the previous research will undoubtedly provide a comprehensive set of information for geneticists and plant breeders to better understand and chalk out the strategies to develop varieties with durable resistance against downy mildew.

## Downy Mildew

### Pathogen and Symptoms

**FIGURE 2:**
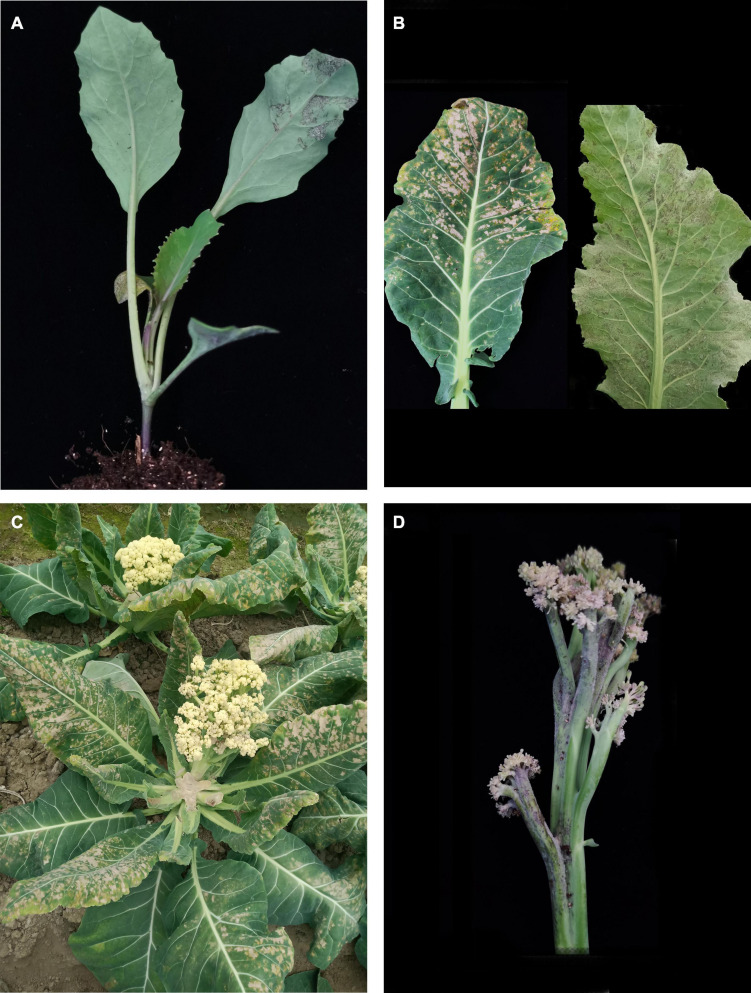
Symptoms of downy mildew infection in cauliflower [**(A)** seedling stage, **(B)** mature leaves during curding stage, **(C)** whole plant during bolting stage, **(D)** stalk of the curd].

Downy mildew is one of the most destructive and commonly occurring diseases of cauliflower caused by *Hyaloperonospora parasitica* Constant (Pers.:Fr) Fr. (formerly *Peronospora parasitica*), an obligate parasite. It is a soil-borne disease and can infect at any growth stage of the crop starting from cotyledon to curd stage (Crute and Gordon, 1987). Downy mildew is prevalent in many cruciferous crop growing countries (Farinhó et al., 2007; Carlier et al., 2012) and causes 50–60% loss in seed production of cole crops (Saha et al., 2020). The characteristics symptoms of downy mildew appear as angular translucid spots in the intervenial spaces of the leaves showing purplish brown on underside and the upper surface looks tan to yellow color (Figure 2). Under favorable conditions, approximately 75–90% of seedling mortality has been reported (Gaikwad et al., 2004). The disease is severe in rainy and cool climates and damages the curds (Bains et al., 1981). The affected curds look brownish at the top, turn dark brown to black later. Cauliflower curd becomes discolored and deformed due to whitish mycelial growth (Dickson and Petzoldt, 1993). In *B. oleracea*, downy mildew infection at the cotyledon stage could cause stunting or death of the seedling (Silue et al., 1996) and also affects the adult plants by reducing yield and head quality (Niu et al., 1983). Various downy mildew symptoms at the seedlings stage, mature leaves during curding stage, whole plant during bolting stage and in the stalk of the curd of cauliflower are depicted in Figures 2A–D, respectively. Due to its systemic nature of infection, several cultural and chemical methods are found to be ineffective to control downy mildew. Using of fungicides also failed to control downy mildew (Vicente et al., 2012) and has led to the evolution of more virulent pathogens (Brophy and Laing, 1992; Singh et al., 2013). Therefore, development of cultivars with inherent resistance to downy mildew could be the viable option for sustainable production of cauliflower.

### Resistance Sources and Genetics of Resistance

Identification of new resistance sources and knowledge of genetics of resistance are prerequisite to develop downy mildew resistant varieties. Two types of resistance; seedling and adult-plant resistance are expressed by downy mildew resistance loci in *B. oleracea*. Several resistance sources were identified in cauliflower and different varieties of *B. oleracea* at the cotyledon and adult-plant stage (Natti et al., 1967; Kontaxis et al., 1979; Singh et al., 1987; Monteirio and Williams, 1989; Thomas and Jourdain, 1990, 1992; Mahajan et al., 1991; Chatterjee, 1993; Sharma et al., 1995; Silue et al., 1995; Jensen et al., 1999a; Pandey et al., 2001; Coelho and Monteiro, 2003b; Carlsson et al., 2004; Branca et al., 2005; Vicente et al., 2012; Singh et al., 2013). Identification of new resistance source should be a continuous process as pathogens evolve and varieties carrying single resistance genes failed to provide resistance against the virulent pathogens. The resistance sources could be used to identify QTLs/genes linked to downy mildew resistance through linkage analysis in cauliflower. Recently, Mohammed et al. (2018) screened some 154 genotypes of *Brassicaceae* family consisting of a wide range of *Brassica* species including *B. oleracea* against a mixture of seven isolates of *Hyaloperonospora brassicae* and identified many highly resistant genotypes. This study highlighted the ready availability of high levels of pathotype-independent resistance across diverse species of *Brassicaceae* against downy mildew paving the way to utilize these resistance sources in vegetable *Brassicaceae* breeding programs where downy mildew is prevalent.

Genetics of resistance to downy mildew was found to be complicated and different mode of inheritance was reported in cauliflower for both seedling and adult-plant resistance. The genetics of seedling resistance varies with resistance source in cauliflower, e.g., single dominant gene (Jensen et al., 1999a), single gene with recessive effect (Hoser-Krauze et al., 1984) and multiple (additive) genes (Hoser-Krauze et al., 1995; Jensen et al., 1999b). Types of genetic control for seedling resistance also varies in other *B. oleracea* varieties; e.g., single dominant gene in *B. oleracea* and broccoli (Natti et al., 1967; Farnham et al., 2002; Vicente et al., 2012), recessive gene(s) in kale, savoy cabbage and brussels sprouts (Carlsson et al., 2004), two dominant independent genes in cabbage (Caravalho and Monteiro, 1996), multiple (additive) genes in broccoli (Jensen et al., 1999b), two duplicate dominant genes in *B. oleracea* var. *tronchuda* (Monteirio et al., 2005) and three to four dominant complementary genes in broccoli (Hoser-Krauze et al., 1995). Resistance at adult-plant stage in cauliflower is governed by single dominant gene (Mahajan et al., 1995; Verma and Singh, 2018; Saha et al., 2020) and recessive epistatic gene (Mahajan et al., 1995). In other varieties of *B. oleracea* also, the genetic control of adult-plant resistance was found to be controlled by a single dominant gene in broccoli (Natti and Atkin, 1960; Barnes, 1968; Coelho and Monteiro, 2003b), cabbage (Barnes, 1968) and in *B. oleracea* var. *tronchuda* (Monteirio et al., 2005).

So, basically the inheritance of resistance to downy mildew at cotyledon and adult-plant stage was found to be under the control of different genetic systems. The resistance loci expressed in the cotyledon stage doesn’t always translate into adult-plant resistance in *B. oleracea* (Dickson and Petzoldt, 1993; Coelho et al., 1998; Coelho and Monteiro, 2003a) as the outcome of resistance could be different in different growth stages. Few accessions of *B. oleracea* were resistant at the cotyledon stage but found as susceptible at adult-plant stage or vice versa (Coelho and Monteiro, 2003a,b). Monteirio et al. (2005) didn’t find any correlation and rejected the hypothesis of ‘the genes conferring cotyledon resistance also confer adult-plant resistance’ while determining the mode of inheritance of cotyledon and adult-plant resistance in *B. oleracea* var. *tronchuda*. The authors reported that cotyledon resistance was controlled by two duplicate dominant genes while adult-plant resistance was governed by a single dominant gene. This makes it difficult to predict the field resistance from cotyledon resistance. Similarly, Coelho and Monteiro (2003a) also observed very poor correlation between the cotyledon and adult-plant resistance in *B. oleracea* and predicted four possible combinations of resistance/susceptibility at cotyledon and adult-plant stages. This could be possible as the two types of resistances are controlled by independent loci. Field adult-plant resistance has high horticultural value compared with the cotyledon resistance (Monteirio et al., 2005). Likewise, the inheritance of downy mildew resistance at four developmental stages was demonstrated to vary slightly during plant development in Chinese cabbage (Zhang et al., 2012). From breeding perspective, seedling resistance being a reliable indicator of adult-plant resistance is more desirable. Genotypes showing resistance at cotyledon stage and not showing resistance at adult-plant stage in the field possess challenge for resistance breeding as adult-plant resistance is logistically more demanding. So, the breeders prefer genotypes having resistance at both the stages of development against downy mildew as this saves considerable amount of time. But in few instances, resistance at the cotyledon stage has been found to be associated with the resistance expressed at the adult stage (Jensen et al., 1999a; Wang et al., 2000). Wang et al. (2000) opined that the selection of resistant broccoli plants at cotyledon stage will also identify plants with high levels of resistance at subsequent developmental stages. Similarly, in *B. napus*, the screening of the cotyledons at the seedling stage provided an accurate estimation of expression of field susceptibilities/resistance for downy mildew (Ge et al., 2008).

Often genetic resistance causes reduction in other components of fitness due to the pleiotropic effect causing genetic fitness cost to *Hyaloperonospora* resistance. This is indicated by a negative correlation between growth/fitness in a disease-free environment and resistance measured under pathogen attack as was observed in *B. rapa*, where 6% slower growth was reported in *Hyaloperonospora*-resistant genotypes than *Hyaloperonospora*-susceptible genotypes in pathogen-free environments (Mitchell-Olds and Bradley, 1996). The authors observed that the resistance genes can have pleiotropic effects on other aspects of plant performance (Simms, 1992).

### Molecular Basis of Downy Mildew Resistance

Among all the *Brassica* species, in Arabidopsis, several downy mildew resistance genes have been characterized. In Arabidopsis, 20 wild-type specificities of resistance to *H. parasitica* (RPP) genes have been identified which are distributed in five chromosomes (Holub, 1997). The RPP genes are NB-LRR type and encode receptor-like proteins containing a conserved nucleotide-binding motif and a leucine-rich repeat domain (Parker et al., 1997; Botella et al., 1998; McDowell et al., 1998; Bittner-Eddy et al., 2000). These RPP genes are the molecular components of the host that are required for genotype-specific recognition of the pathogen during the interaction between Arabidopsis and *H. parasitica*.

The RPP genes include single locus downy mildew resistance gene *RPP13* and a complex locus *RPP1*. The simple locus *RPP8/HRT1* is also another example in which different functional alleles were found conferring resistance to widely divergent pathogens (McDowell et al., 1998; Cooley et al., 2000). The resistance gene *RPP5* was characterized and found to be inherited as a single locus by Parker et al. (1993). Positional cloning of the *RPP5* locus (Parker et al., 1997) was the first step in understanding the recognition specificity of plant-pathogen interactions at the molecular level. Downy mildew resistance genes vary in the process of conferring defense via different regulatory proteins (Aarts et al., 1998; Eulgem et al., 2004). For e.g., a single downy mildew resistance gene *RPP7* could confer accumulative; both salicylic acid dependent and independent defense responses (McDowell et al., 2000; Tor et al., 2002; Eulgem et al., 2007). ‘Gene-for-gene’ hypothesis was established in the *At-HpA* pathosystem. This became evident when an outcross of HpA enables five independent At-recognized effectors (ATR1, ATR4, ATR5, ATR8, and ATR13) corresponding to different cloned downy mildew resistance genes (Gunn et al., 2002).

Mutational approach was adopted to identify the genes that are necessary for downy mildew resistance mediated by RPP genes. In Arabidopsis, several wild-type genes were identified by mutational analysis which is required for R gene-mediated resistance (Glazebrook et al., 1997). An RPP-non-specific locus called EDS1 (for enhanced disease susceptibility) was revealed by mutational approach and was found to be a necessary component of the resistance response for several RPP genes which function in the upstream from the convergence of disease resistance pathways in Arabidopsis (Parker et al., 1996). Aarts et al. (1998) revealed a strong requirement of EDS1 by several R gene loci (*RPP2*, *RPP4*, *RPP5*, *RPP21*, and *RPS4*) conferring resistance to *H. parasitica* in Arabidopsis and the mutation in EDS1 abolished resistance conferred by several RPP loci (Parker et al., 1996). However, another RPP locus, *RPP8* doesn’t exhibit any strong requirement for EDS1 for isolate-specific resistance to *H. parasitica*.

### Cloning of Major Downy Mildew Resistance Genes

Isolation and cloning of resistance gene, called map-based or positional cloning is an important strategy for the isolation of ‘R’ gene which started in early 1990 (Parker et al., 1993). Majority of ‘R’ genes are present in larger or smaller clusters instead of randomly distributed in the chromosomes. These are called major recognition gene complexes (MRC) loci. About 19 downy mildew resistance RPP genes are grouped in to three MRCs and four others are scattered on chromosomes 1 and 2 of *Arabidopsis thaliana*. Though so many RPP genes have been postulated, a few have been cloned. *RPP5 was* the first downy mildew resistance gene to be cloned on chromosome 4 of *Arabidopsis thaliana* (Parker et al., 1997) which encoded TIR-NBS-LRR receptor-like proteins. This was followed by the cloning of *RPP1* and *RPP8* genes (Botella et al., 1998; McDowell et al., 1998). The multicopy locus *RPP1* found to contain several downy mildew resistance genes of TIR-NBS-LRR subclass type which differ in specificity (Botella et al., 1998). *RPP8* locus could be studied for how recombination slippage and domain shuffling had led to new recognition specificities (McDowell et al., 1998; Cooley et al., 2000). *RPP13* gene was cloned (Bittner-Eddy et al., 1999) and it turned out that *RPP13* and *RPP11* were allelic and mapped to the same locus on chromosome 3 of *Arabidopsis thaliana* (Joos et al., 1996). But both the genes recognized different pathogen avirulence determinants (Bittner-Eddy et al., 2000). Several other downy mildew resistance genes namely, *RPP2A/RPP2B* (Sinapidou et al., 2004), *RPP4* (Van der Biezen et al., 2002) were also cloned. Additionally, another adult-plant resistance gene, *RPP31* was mapped on chromosome 5 in *Arabidopsis thaliana* (McDowell et al., 2005). But till date, limited attempts were made in cauliflower and *B. oleracea* to isolate and clone the downy mildew resistance genes.

### Advances in Molecular Breeding Research for Downy Mildew Resistance

**FIGURE 3:**
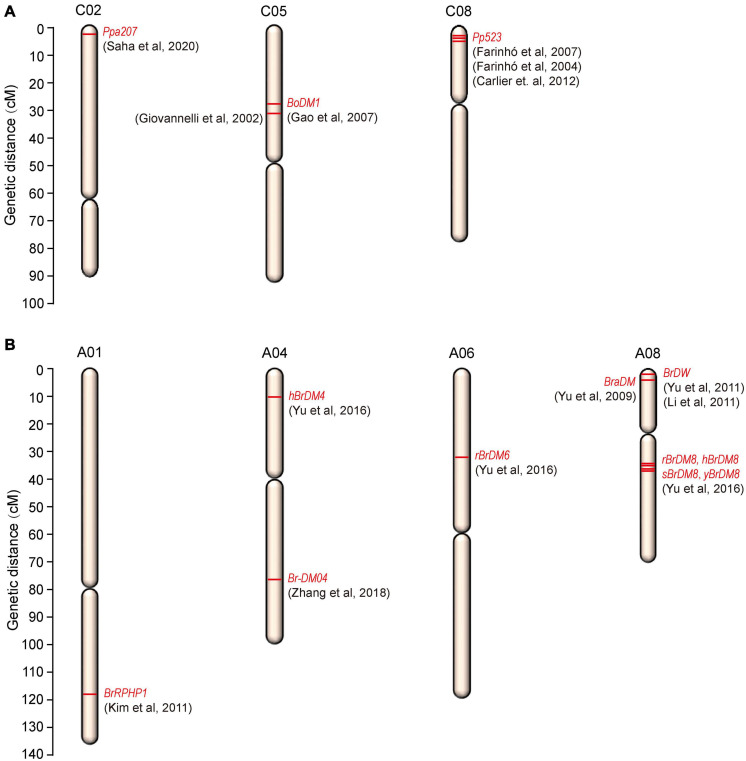
Chromosomal locations of downy mildew resistance loci mapped on **(A)** *B. oleracea* and **(B)** *B. rapa*.

The resistance to downy mildew is most likely to be QTL-specific and the QTLs are primarily associated with the developmental stages of the plant. So, for downy mildew resistance breeding, identification of QTLs at both the developmental stages will be much useful. Though in the recent years, advances were made in genetic studies for downy mildew resistance, only few major ‘R’ genes and QTLs have been identified in cauliflower and *B. oleracea* (Table 1 and Figure 3A).

**TABLE 1:** List of genes/QTLs associated with Downy mildew resistance in *Brassica oleracea* L.

Disease	Species	Mapping population	Gene locus/QTL	Chr/LG	Linked Marker	References
Downy mildew (*Hyaloperonospora parasitica*) Constant (Pers.:Fr) Fr.	*Brassica oleracea* var. *italica*	F2		C05	UBC359620 and OPM16750	Giovannelli et al. (2002)
	*Brassica oleracea* var. *italica*	F2	*Pp523*	C08	OPK17_980 and ATCTA_133/134	Farinhó et al. (2004)
	*Brassica oleracea*	F2	*Pp523*	C08	SCR15 and SCAFB1/BfuI	Farinhó et al. (2007)
	*Brassica oleracea*	F2	*BoDM1*	C05	DM1-F and DM1-R	Gao et al. (2007)
	*Brassica oleracea*		*Pp523*	C08	CB10139 and CB10028	Carlier et al. (2012)
	*Brassica oleracea* var. *botrytis*	F2	*Ppa3*	−	OPC141186, OPE141881, ISSR-231103	Singh et al. (2012)
	*Brassica oleracea* var. *botrytis*	F2	−	−	OPC141186, OPE141881, ISSR-231103	Singh et al. (2015)
	*Brassica oleracea* var. *botrytis*	RIL	*Ppa207*	C02	BoGMS0486 and BoGMS0900	Saha et al. (2020)

In cauliflower, a single dominant R gene, *Ppa3* was mapped for adult-plant resistance against downy mildew (Singh et al., 2012). Two RAPD (OPC141186 and OPE141881) and one ISSR (ISSR-231103) marker were found in linkage with the resistance gene, *Ppa3* by bulk-segregant analysis. But the markers identified were located far apart (22.3, 10.6, and 26.4 cM) from the *Ppa3* gene raising the doubt about its utility in marker-assisted selection for downy mildew resistance. Singh et al. (2015) again reported the same RAPD (OPC141186 and OPE141881) and ISSR (ISSR231103) markers to be linked to downy mildew resistance in cauliflower. Very recently, Saha et al. (2020) identified a new monogenic dominant downy mildew resistance gene *Ppa207* on chromosome 2 of cauliflower. The new resistance gene was mapped at 4.8 cM interval on linkage group 2 (C02) of cauliflower and was flanked by two SSR markers, BoGMS0486 and BoGMS0900 at a distance of 3.6 and 1.2 cM, respectively, from *Ppa207*. The 4.8 cM marker interval corresponds to a physical distance of 20.3 Mb and the two markers BoGMS0486 and BoGMS0900 were located at 2.9 and 23.2 Mb positions, respectively, on chromosome C02. The identification of this new gene and the linked markers will be very useful in molecular breeding research to develop downy mildew resistant varieties. In other *B. oleracea* crops, few QTLs and linked markers were also reported for downy mildew resistance. In broccoli, the disease resistance genes have been tagged by different authors previously (Agnola et al., 2000; Farinho et al., 2000). One locus conferring resistance at cotyledon-stage in broccoli was mapped and two SCAR markers derived from two RAPD fragments (UBC359620 and OPM16750) were found linked in coupling to the resistance locus at 6.7 and 3.3 cM from the resistance locus (Giovannelli et al., 2002) and this finding has led to the identification of some putative resistance gene homologues by Gao et al. (2003). This mapped locus conferring downy mildew resistance in broccoli was located in close linkage to the glucosinolate pathway gene *BoGsl-elong* on a high-density map of *B. oleracea* (Gao et al., 2007). A dominant and monogenically inherited resistance locus was identified in broccoli responsible for adult-plant resistance (Coelho and Monteiro, 2003b) which was later named as *Pp523*. Bulk-segregant analysis mapped this resistance locus *Pp523* to linkage group 3 (LG3) in broccoli and two markers (OPK17_980 and ATCTA_133/134) were found linked in coupling to the resistance gene and located at a distance of 3.1 and 3.6 cM, respectively, at each side of the resistance gene (Farinhó et al., 2004). This resistance locus located in LG3 was later assigned to the chromosome C8 of *B. oleracea* by Carlier et al. (2012) and in addition to the previously mapped markers, two additional SSR markers; CB10139 and CB10028 were found flanking the locus at a distance of 2.4 and 6.8 cM, respectively. So, enrichment of the chromosome C8 can be used as a tool for the map-based cloning of *Pp523*. SCAR and CAPS markers flanking this resistance locus, *Pp523* were identified by Farinhó et al. (2007). Due to the widespread colinearity and presence of conserved genomic sequences between *B. oleracea* and *Arabidopsis thaliana* (Lan et al., 2000; Babula et al., 2003), markers surrounding the *Pp523* locus were searched against the Arabidopsis genome and were found in synteny with the top arm end of chromosome 1 of *Arabidopsis thaliana* (Farinhó et al., 2007). This information may help in the construction of a fine-scale map of the corresponding genomic region in *B. oleracea*. When the genome of *B. oleracea* was not sequenced, physical mapping of the gene of interest was being carried out via construction of a contig of large insert DNA clones, usually BACs. The conserved synteny between *B. oleracea* and *Arabidopsis thaliana* was exploited to carry out the physical mapping of the resistance locus *Pp523* via construction of BAC libraries as physical identification of the genomic region is prerequisite to map-based cloning (Carlier et al., 2011). After screening of the BAC libraries, the selected BAC clones were mapped to three different genomic regions of *B. oleracea*. 83 BAC clones were accurately mapped within a 4.6 cM region surrounding the downy mildew resistance locus *Pp523*, but a subset of 33 BAC clones were mapped to aprrox. 60 cM away from the resistance gene on chromosome C8 and a subset of 63 BAC clones were mapped to chromosome C5 reflecting triplication of the *Brassica* genomes since their divergence from a common ancestor.

Though in *Arabidopsis thaliana*, map-based or positional cloning strategy was used to isolate several downy mildew resistance genes (discussed earlier), enough attention was not paid to isolate the downy mildew ‘R’ genes in *B. oleracea.* As there is a prevalence of conserved genomic regions between *B. oleracea* and *Arabidopsis thaliana*, comparative genome analysis (Yu et al., 2014) and pan-genome analysis (Golicz et al., 2016) may lead to the identification of orthologous genes in *Brassica* species which may be useful for breeding of downy mildew resistance.

In *B. rapa*, a closely related species of *B. oleracea*, several major downy mildew resistance QTLs and candidate genes are reported (Yu et al., 2009, 2011, 2016; Kim et al., 2011; Li et al., 2011; Zhi et al., 2016; Zhang B. et al., 2018) for downy mildew disease (Table 2 and Figure 3B). Four major QTLs (*sBrDM8*, *yBrDM8*, *rBrDM8*, and *hBrDM8*) for downy mildew resistance at the seedling, young plant, rosette, and heading stages were mapped on A08 chromosome of Chinese cabbage (Yu et al., 2016) which were identical to another major QTL, *BraDM*, identified previously at the seedling stage by Yu et al. (2009). Also, two minor QTLs, *rBrDM6* (A06) and *hBrDM4* (A04), were found active at the rosette and heading stages. Interestingly, the outcome of this research indicated that the major QTLs on A08 provide effective downy mildew resistance at every developmental stage and also confirmed that the genetic resistance to downy mildew in seedlings and adult plant is somewhat different. Additionally, a serine/threonine kinase family gene was identified as the possible candidate gene (*Bra016457*) and importantly, the diagnostic SNP markers (A08-709, A08-028, and A08-018) identification was found to be effective when used in MAS to breed for downy mildew resistance in *B. rapa*. A downy mildew resistance QTL, *Br-DM04* was mapped in a region of 2.7 Mb on chromosome A04 of *B. rapa* and was fine mapped to a 160-kb region, between the SNP markers A04_5235282 and A04_5398232 containing 17 genes encoding proteins (Zhang B. et al., 2018). Based on sequence annotations for these genes, four candidate genes, *BrLRR1, BrLRR2, BrRLP47*, and *BrRLP48* related to disease resistance were identified. RT-PCR analysis showed the up-regulated expression of *BrRLP48* in response to downy mildew inoculation or salicylic acid (SA) treatment in the resistant line MM. These findings led the authors to conclude that the disease-inducible expression of the resistance gene, *BrRLP48*, contributed to downy mildew resistance.

**TABLE 2:** List of genes/QTLs associated with Downy mildew resistance in related *Brassica* species.

Disease	Species	Mapping population	Gene locus/QTL	Chr/LG	Linked marker	References
Downy mildew (*Hyaloperonospora parasitica*) Constant (Pers.:Fr) Fr.	*Brassica rapa* ssp. *pekinensis*	DH	*BraDM*	A08	K14-1030, PGM, Ol12G04	Yu et al. (2009)
	*Brassica rapa* ssp. *pekinensis*	F2	*BrRPHP1*	A01	OPA08650 and BrPEK15B	Kim et al. (2011)
	*Brassica rapa* ssp. *pekinensis*	DH	*BrDW*	A08	Brb062-Indel230, Brb094-DraI787, Brb094-AatII666, Brb043-Bg1II715, Brh019-SNP137, bru1209	Li et al. (2011)
	*Brassica rapa* ssp. *pekinensis*	DH	*BrDW*	A08	SCK14-825, kbrb058m10-1, kbrb006c05-2	Yu et al. (2011)
	*Brassica rapa* ssp. *pekinensis*	DH	*sBrDM8, yBrDM8, rBrDM8, hBrDM8, rBrDM6, hBrDM4*	A08, A06, A04	A08-709, A08-028, A08-018	Yu et al. (2016)
	*Brassica rapa* ssp. *pekinensis*	Inbred lines	*QTL*	A01	A0124655323	Zhi et al. (2016)
	*Brassica rapa* ssp. *pekinensis*	DH, F2	*Br-DM04*	A04	A04_5235282 and A04_5398232	Zhang B. et al. (2018)

Also, in radish, an edible root vegetable of the family *Brassicaceae*, a single dominant locus; *RsDmR* was identified and mapped at the seedling stage (Xu et al., 2014). One SRAP marker Em9/ga24370 was the most tightly linked one with a distance of 2.3 cM to *RsDmR*. In cucurbits especially cucumber, several downy mildew resistance genes have been mapped. In one of the important studies, Berg et al. (2020) analyzed one QTL, *DM4.1* on chromosome 4 for downy mildew resistance in cucumber which consisted of three subQTLs (*DM4.1.1, DM4.1.2, and DM4.1.3*). Transcriptome analysis revealed that many defense pathway genes were differentially expressed in NIL DM4.1.1/.2. Fine mapping revealed the RLK gene *CsLRK10L2* as a likely candidate for subQTL *DM4.1.2* causing a loss-of-function mutation in the susceptible parent HS279. Two major QTLs and two QTL hotspots were identified for downy mildew resistance in cucumber using an F_2_ population (Innark et al., 2020). In another study, QTL mapping in a RIL population of cucumber identified 11 QTLs for downy mildew resistance accounting for more than 73.5% of total phenotypic variance (Wang et al., 2018). Among 11 QTLs, *dm5.1*, *dm5.2*, and *dm5.3* were the major-effect contributing QTLs, whereas *dm1.1*, *dm2.1*, and *dm6.2* conferred susceptibility. Additionally, three powdery mildew resistance QTLs, *pm2.1*, *pm5.1*, and *pm6.1* were found co-localized with downy mildew resistance QTLs, *dm2.1, dm5.2*, and *dm6.1*, respectively. Through BSA-seq approach, Win et al. (2017) mapped five QTLs (*dm2.2, dm4.1, dm5.1, dm5.2, and dm6.1*) in cucumber and *dm2.2* showed the largest effect on downy mildew resistance. Yoshioka et al. (2014) revealed that high resistance of a cucumber progeny line CS-PMR1 was associated with many QTLs with relatively small effects, whereas the moderate resistance of Santou, an old Japanese cultivar was associated with one major QTL. The authors opined that the identified QTLs may assist in the selection to breed new cultivars efficiently carrying adequate levels of downy mildew resistance. Zhang et al. (2013) observed the quantitative resistance to downy mildew in cucumber inbred line, K8 and detected five resistance QTLs (*dm1.1, dm5.1, dm5.2, dm5.3*, and *dm6*) on chromosome 1, 6 and 5. Interestingly, six and four nucleotide binding site (NBS)-type resistance gene analogs (RGAs) were predicted in the region of *dm5.2* and *dm5.3*, respectively.

### Mechanism of Downy Mildew Resistance

The plants have evolved different mechanisms to counteract the pathogen attack and different defense-related gene families such as nucleotide-binding site (NBS), receptor-like kinase (RLK) protein, receptor-like protein (RLP), mitogen-activated protein kinase (MAPK) and phytohormones such as salicylic acid (SA), jasmonic acid (JA), ethylene (ET), abscisic acid (ABA) are involved in the innate immunity system (Boller and Felix, 2009; de Jonge et al., 2012; Yang et al., 2013; Liu W. et al., 2014; Jiang et al., 2018). Though some downy mildew resistance loci have been identified, the molecular mechanism of downy mildew resistance remains poorly understood in *B. oleracea* though few reports are available in *B. rapa* (Yu et al., 2016; Zhang et al., 2018). In Arabidopsis, attempts were made to understand the mechanism of downy mildew resistance. Powdery Mildew Resistant 4 (PMR4) and Rab GTPase homolog 4c (RabA4c) were involved in the callose deposition in Arabidopsis leading to downy mildew resistance, and also some genes of SA and JA signaling pathways such as PRs, NPRs, and WRKYs were reported to play roles in downy mildew resistance (Caillaud et al., 2013; Coker et al., 2015). Again, the expression patterns of the major genes of SA and JA signaling pathway confirmed SA as the major plant hormone involved in downy mildew resistance in *B. rapa* (Gao et al., 2014; Chen et al., 2015). A comparative transcriptome analysis of downy mildew infected Chinese cabbage lines identified differentially expressed candidate genes involved in the plant-pathogen interaction pathway (Zheng H. et al., 2020) and the proteins encoded by these genes were reported to play an important role in the pattern-triggered immunity (PTI) and effector triggered immunity (ETI) processes of some model plants such as Arabidopsis, rice, tobacco, and tomato. The authors demonstrated the involvement of proteins processing in endoplasmic reticulum and circadian rhythm pathways in the resistance mechanisms against downy mildew. Furthermore, the down-regulation of photosynthetic genes was reported during *H. brassicae* infection which was in line with the results of Xiao et al. (2016) who reported the down-regulation of energy metabolism genes, particularly those involved in the photosynthetic carbon cycle (PCC), providing protection to Chinese cabbage against *H. parasitica*. So, this indicated that energy metabolism is playing a role in the resistance against downy mildew during pathogen invasion in *Brassica* crops.

Whole genome-wide gene expression profiles may help gain insight in to the resistance mechanism against downy mildew. Li et al. (2018) studied the genome-wide gene expression profiles in Chinese cabbage and listed the immunity-related genes. To further improve the understanding of the resistance mechanism against downy mildew at the transcriptional level, the role of long non-coding RNAs were explored in B. *rapa*. Long non-coding RNAs (lncRNAs) are types of ncRNAs with a length of more than 200 nucleotides (Cabili et al., 2011). Some defense related lnc RNAs have been identified which are found to be regulating the expression of many resistance genes in several crop such as powdery mildew in melon (Gao et al., 2020), turnip crinkle virus infection in Arabidopsis (Gao et al., 2016), *Phytophthora infestans* in tomato (Cui et al., 2017, 2019; Hou et al., 2020) and *Verticillium dahliae* and *Botrytis cinerea* in cotton (Zhang L. et al., 2018). For the first time, Zhang et al. (2021) carried out the high-throughput RNA sequencing and analyzed the disease responding mRNAs and long non-coding RNAs in two resistant (T12–19 and 12–85) and one susceptible line (91–112). Different expression pattern and the proposed functions of the differentially expressed non-coding RNAs among the resistant and susceptible lines indicated that each has a distinct disease response mechanism. More cis and trans-functional long non-coding RNAs were found in the resistant lines than the susceptible line which regulates the genes involved in disease defense response. A candidate related long non-coding RNA, STRG.19915 was identified which is a long non-coding natural antisense transcript of a MAPK gene, *BrMAPK15*. MSTRG.19915-silenced seedlings showed enhanced resistance to downy mildew which could be attributed to the up-regulated expression of *BrMAPK15*. This important research laid the foundation for future studies to decipher the mechanism of downy mildew resistance in *Brassica* crops.

### Multi-Loci Molecular Polymerization Breeding

Polymerization breeding is a technique used in plant breeding in which favorable genes from different lines are integrated into a cultivated variety through genetic engineering, hybridization, backcrossing, and multiple cross (Yadav et al., 1990; Servin et al., 2004). Mostly, the crop-breeding strategies for resistance to biotic and abiotic stresses are based on deploying only single resistance gene into the plants which is not durable and short-lived (McDonald and Linde, 2002; Steiner et al., 2019). Therefore, combining multiple *R* genes from different sources into a single plant by gene pyramiding would increase the durability of disease resistance and will help in building an ideal genotype. Furthermore, combining genes with different but complementary resistance spectra could provide gene pyramids against a broad-spectrum of pathogenic races (Sanchez-Martin and Keller, 2019). Combining resistance genes based on marker-assisted selection could accelerate gene pyramiding by identifying and selecting plants with desirable allele at a very early stage and was found to be proficient and economical and is a simple technique to build multiple stress tolerance in crops (Chukwu et al., 2019; Angeles-Shim et al., 2020).

Marker-assisted gene pyramiding of resistance QTLs have been successfully applied in several crops consequently producing a number of varieties and lines with improved resistances against biotic stresses, such as late blight, bacterial blight, gall midge, mosaic viruses, powdery mildew and many abiotic stresses, such as salinity, drought, heat, and cold with a higher yield and desired nutritional quality (reviewed by Dormatey et al., 2020). Especially, the multi-loci polymerization resistance breeding is a success story in rice where plant scientists successfully utilized this technique to accomplish durable resistance against various diseases such as gall midge (Das and Rao, 2015), blast (Jamaloddin et al., 2020), brown planthopper (Liu et al., 2016), bacterial blight (Singh et al., 2001; Joseph et al., 2004; Jamaloddin et al., 2020; Ramalingam et al., 2020), sheath blight (Ramalingam et al., 2020), blast and bacterial blight (Narayanan et al., 2002), bacterial, sheath blight and stem borer (Datta et al., 2002) etc. Wu et al. (2019) identified 37 SNP polymerization regions for 36 agronomic traits in 287 pepper accessions which could be the focus of molecular marker development to improve the efficiency of multi-trait pyramid breeding. Though, the multi-loci gene pyramiding has been fully utilized in several crops, the technique has not been fully utilized in *B. oleracea* for biotic stress resistance. But in *B. rapa* and *B. napus*, several authors have attempted to pyramid multiple resistance loci for clubroot (Matsumoto et al., 2012; Shah et al., 2019) and nematode resistance (Zhong et al., 2019) by polymerization of several identified resistance loci/genes by marker-assisted selection. Shah et al. (2019) pyramided two clubroot resistance genes (*CRb* and *PbBa8.1*) through marker-assisted selection and developed homozygous lines which showed strong resistance against a number of *P. brassicae* field isolates compared with the heterozygous pyramided and single resistant homozygous lines of *B. napus*. But no polymerization breeding strategy has been adopted for resistance against downy mildew resistance. As discussed, several downy mildew resistance QTLs specific to seedling and adult plant stage have already been identified by different researchers in *B. oleracea* which could be pyramided to confer durable resistance. The pyramiding of seedling and adult-plant resistance QTLs will provide durable resistance since the QTLs will act against downy mildew at different developmental stages of the plant as was done for leaf rust resistance in wheat (Samsampour et al., 2010) wherein both seedling resistance gene *Lr25* and adult-plant resistance gene *Lr48* were pyramided together through marker-assisted selection. Similarly, in apple, pyramiding of three resistance QTLs to scab resulted in durable resistance which acted at different stages of fungal infection cycle (Laloi et al., 2017). Marker-assisted gene pyramiding by combining two or more downy mildew resistance genes was performed in several crops such as in grape vine (Schwander et al., 2012; Nascimento-Gavioli et al., 2017; Saifert et al., 2018) and sunflower (Qi et al., 2017; Qi and Ma, 2019). Pyramiding of QTLs with different specificities/broad-spectrum QTLs, and QTLs associated with different resistance mechanism are expected to increase the durability by showing stable levels of resistance in multiple environments. Breeders also have to reflect the number of resistance loci to be pyramided based on the degree of resistance contributed by different loci. As in *B. oleracea* and *B. rapa*, most of the QTLs reported for downy mildew resistance are of major effects, combining these QTLs in the background of susceptible elite variety is expected to increase the durability which could be accomplished via a breeding strategy through the polymerization of multiple loci by marker-assisted gene pyramiding.

### Gene-Editing in Resistance Breeding

Susceptibility of crops to a multitude of pathogens and pests including viruses, bacteria, oomycetes, fungi, insects, nematodes, etc., poses a major challenge to the plant researchers to enhance the resistance and improve the productivity as well. Several innovative gene-editing techniques such as engineered endonucleases/meganucleases (EMNs), zinc-finger nucleases (ZFNs), TAL effector nucleases (TALENs), and CRISPR-associated protein 9 (CRISPR/Cas9) have emerged as important tools to engineer disease resistant crops (Borrelli et al., 2018; Langner et al., 2018; Dong and Ronald, 2019; Mushtaq et al., 2019). But the main concern for deploying any gene-editing approach for broad-spectrum and durable resistance are; (i) the fundamental knowledge about which gene(s) to modify and (ii) which type of modification to perform in these genes (Miladinovic et al., 2021). Though targeting a single resistance gene for inactivation is technically less challenging (Borrelli et al., 2018), durable resistance could be achieved by targeting several resistance genes, multiple metabolic and immune pathways induced downstream of NLRs whose resistance are more difficult to break down (Miladinovic et al., 2021). The steadily growing knowledge about the molecular mechanism of plant-pathogen interactions may facilitate the deployment of gene-editing technologies to manipulate the disease resistance genes. Among all the gene-editing techniques, the application of CRISPR/Cas9 technology for disease resistance is the most sought approach to improve crop resistance to pathogens (Borrelli et al., 2018). Now that draft genome sequences of most of the crops are available, gene-editing could be applied to all the target genes to mutate it to get the desired phenotype even in a complex genome like allotetraploid *B. napus* (Braatz et al., 2017). CRISPR/Cas9 is a highly promising system for gene-editing and gained popularity due to its precise specificity, multigene-editing, minimal off-target effects, higher efficiency and simplicity (Kumar and Jain, 2015). CRISPR/Cas9 has been proved as a fascinating tool in plant breeding technique and has revolutionized the development of various disease resistance cultivars against a broad-spectrum of pathogens by precise modification of the genes that confer susceptibility to a given pathogen. Importantly, the mutation generated from CRISPR/Cas9 system is stable and heritable which could be segregated from Cas9/sgRNA helping in the development of transgene-free progeny in only one generation (Zhang and Zhou, 2014; Zhang et al., 2014). After this significant breakthrough of the 20th century, the technique has been used for alteration of plant immunity at several stages of different crops. Additionally, many administrative agencies including the USDA (United States) and the Canadian Food Inspection Agency (Canada) don’t consider the genome-edited crops as GMOs, thus providing a greater opportunity for them to enter the market in much lower regulatory costs (Callaway, 2018; Schmidt et al., 2020).

Recent advances have been made in the area of CRISPR technique in different crops to generate varieties that are resistant against several pathogens. For example, the technique has been used successfully for powdery mildew resistance in wheat (Wang et al., 2014) and tomato (Nekrasov et al., 2017), against bacterial blight in rice (Oliva et al., 2019), geminivirus infection in Arabidopsis and *N. benthamiana* (Ji et al., 2015), for citrus canker resistance in citrus (Peng et al., 2017), *Phytophthora infestans* resistance in potato (Andersson et al., 2017), and *Verticillium dahliae* resistance in upland cotton (Zhang Z. et al., 2018) etc. The readers are referred to the excellent reviews written by several authors mentioning about the application of CRISPR technology to improve resistance to biotic stresses in different crops (Borrelli et al., 2018; Langner et al., 2018; Yin and Qiu, 2019; Ahmar et al., 2020; Schenke and Cai, 2020).

In recent years, the application of different gene-editing techniques in several horticultural crops has substantially increased to enhance crop production and quality (Xu et al., 2019). Among the horticultural crops, most of the gene-editing studies (72%) have been conducted in vegetables (Xu et al., 2019) and among the vegetables, tomato has gained more attention for gene-editing studies (Wang et al., 2019; Salava et al., 2021) which have been performed most frequently up to 42% (Xu et al., 2019). Recently, the mutation of a single gene, *DMR6* (downy mildew resistance 6) in Arabidopsis generated plants with increased salicylic acid levels and showed resistance to the bacterium *Pseudomonas syringae* and oomycete *Phytophthora capsici* (Zeilmaker et al., 2015). Interestingly, the tomato ortholog of *SlDMR6-1* was targeted using CRISPR/Cas9 system and the mutants showed resistance to *Pseudomonas syringae* pv. tomato, *Phytophthora capsica* and *Xanthomonas* spp. without any significant detrimental effects on tomato growth and development (de Toledo Thomazella et al., 2016). Knocking out of *DMR6* was found to be a promising strategy in conferring broad-spectrum resistance to plants.

The first gene-editing in a horticultural crop was reported in *B. oleracea* which was achieved via a TALEN (Sun et al., 2013). After this first report, in the following years, significant progress has been made in *Brassica* crops, especially in *B. napus* (Chang et al., 2021) targeting several genes such as yield related genes (Braatz et al., 2017; Yang et al., 2017; Zhai et al., 2019), quality related genes (Okuzaki et al., 2018; Zhang et al., 2019), genes related to plant architecture (Zheng M. et al., 2020), seed coat color related genes (Zhai et al., 2020) and disease resistance genes (Sun et al., 2018). But the function of the genes targeted by gene-editing in *Brassica* crops have mostly focused on the genes affecting development, metabolism and not much attention has been paid to target biotic stress-response genes except a few. In Arabidopsis, *AtWRKY11* and *AtWRKY70* genes were involved in JA and SA induced resistance to pathogens whereas in *B. napus*, *BnaWRKKY11* and *BnaWRKKY70* genes were found to be differentially expressed after inoculated with *Sclerotinia sclerotiorum*. Two Cas9/sgRNA constructs targeting both the genes generated mutants and it was found that the *BnaWRKKY11* mutant showed no significant difference to *Sclerotinia* resistance compared to the wildtype whereas *BnWRKY70* mutants demonstrated better resistance (Sun et al., 2018). The results indicated that *BnWRKY70* could act as a regulating factor in negatively controlling the Sclerotinia resistance. In *B. oleracea*, RNA-guided Cas9 system was used to generate targeted and heritable mutations showing enough potential for rapid characterization of gene function (Lawrenson et al., 2015). CRISPR/Cas9 was used to target *BoPDS* gene in cabbage and 37.5% of the transgenic cabbage shoots carried *BoPDS* gene mutations as a result of nucleotide deletions at the expected position demonstrating CRISPR/Cas9 system as a powerful tool for cabbage variety improvement (Ma et al., 2019). Very recently, Cao et al. (2021) edited the *BoCER1* gene related to glossy phenotype in cabbage by CRISPR/Cas9 and three plants with edited genomes exhibited reduced wax content compared with the wildtype. Except these few, there are no reports on gene-editing thus far in *B. oleracea* varieties especially targeting biotic stress-response genes in spite of having high frequency of regeneration. Nevertheless, gene-editing by CRISPR/Cas9 is going to address the challenges arising due to the evolving nature of pathogens. So, in near future, we can expect that targeted gene-editing through CRISPR-Cas9 will become increasingly indispensable to develop plant varieties that are resilient to a wide range of biotic stresses.

## Challenges

Although it is easier to deploy race-specific ‘R’ genes compared to polygenic resistance in resistance breeding, identification of ‘R’ genes remains a challenge due to the genome complexity of *Brassica* species. The triplication of the *Brassica* genome (Cavell et al., 1998; Lagercrantz, 1998; Lan et al., 2000; O’Neill and Bancroft, 2000; Parkin et al., 2005) revealed the complexity of *Brassica* genome organization. Triplication of *Brassica* genome has led to the clustering and duplication of ‘R’ genes making its identification a challenging task as these genes tend to collapse in genome sequence assemblies. The ‘R’ gene clustering against downy mildew disease in *B. oleracea* (Carlier et al., 2011) is the consequence of *Brassica* genome complexity. In *B. oleracea*, a single dominant resistance gene locus, *Pp523* conferring resistance to downy mildew was mapped to two regions of chromosome C8 and C5 reflecting the triplication of chromosome regions (Carlier et al., 2011). Apart from downy mildew, the evidence of complexity of *Brassica* genome organization has been confirmed in the case of other resistance genes such as clubroot resistance. In *B. napus*, same genomic region was acting both as a race-specific major gene and as a QTL region with quantitative resistance against different pathotypes of clubroot disease (Manzanares-Dauleux et al., 2000; Rocherieux et al., 2004). The genome complexity of *Brassica* genome is one of major bottleneck in ‘R’ gene identification. So it is not reliable to depend on the single reference genome for resistance gene identification, instead need to explore the pan-genomes to identify the novel ‘R’ genes.

In addition to genome complexity, *B. oleracea* lacks sufficient resistance sources for downy mildew and the resistance level vary with different varieties of *B. oleracea*. Moreover, in *B. oleracea*, especially cauliflower has a narrow genetic diversity (Tonguç and Griffiths, 2004; Zhao et al., 2015; Zhu et al., 2018); necessitating greater exploration of the related wild species to introgress novel resistance genes. Instead of breeding materials and cultivars, diverse genetic resources should be used in the genetic mapping studies to broaden the genetic base for resistance breeding.

The presence of high genetic variation in different isolates/pathotypes of downy mildew (Choi et al., 2003) in *Brassica* poses substantial problem in identifying resistance against the pathotypes showing different pathogenecity. This leads to the difficulties in identifying broad-spectrum resistance QTLs against downy mildew. Both the host-specificity and non-host specificity exhibited by different genotypes of the same host causing varied resistance ranging from intermediate to complete resistance could be the strategy to control the diverse pathotypes. The detailed study of the genetic basis of several resistance levels present in the pathosystem and host-pathogen interaction would also throw some limelight in this direction. Often, cultivating one kind of genotypes containing a particular resistance gene may impact the stability of ‘R’ genes due to the selection pressure on a particular pathogen. So, the breeders should avoid growing a single type of cultivar containing a single dominant resistance gene for years together.

Inappropriate pathotype/race classification of pathogen isolates also promotes improper ‘R’ gene identification while classifying qualitative or quantitative resistance. Though the pathotype classification had been carried out in downy mildew (Natti, 1958), the application of host differentials became obscured as different host differentials are not available across countries leading to its limited use and is confined to a particular locality. Moreover, as a consequence of pathogen evolutionary process, pathogens evolve and over time, the breakdown of resistance occurs. So, new set of host differentials need to be updated and validated for proper classification of future pathotypes which should be a continuous process. Efforts were made for an additional host differential for downy mildew in *B. oleracea* (Coelho et al., 2012) using the European isolates. A world database for each pathogen need to be created through international collaboration like the initiative was taken to resolve the blackleg nomenclature issue (Plissonneau et al., 2017) at the *Brassica* 2016 conference. Application of different scoring methods may also complicate the process of proper identification of R genes. In downy mildew, at least three different scoring systems have been applied by several researchers (Dickson and Petzoldt, 1993; Wang et al., 2000). Additionally, a robust race/pathotype differentiation system should be in place to separate different and similar races/pathotypes. Though for downy mildew, there is a lack of proper race differentiation system, Goodwin et al. (1990) had applied a pathogen isolate differentiation system to *H. parasitica* isolates to confirm host differentials which would be highly beneficial for race differentiation.

## Future Perspective and Strategies

For a successful molecular breeding program, diverse resistance sources should be available for the introgression of resistance genes. So, the wild relatives of cultivated *B. oleracea* need to be tapped for better resistance against downy mildew. The screening of related *Brassica* species and land races should be performed extensively to identify novel and superior resistance alleles. Introgression of R genes can be accomplished by wide-hybridization between divergent groups in the *Brassicaceae* via interspecific or intergeneric hybridization.

To predict the durability of resistance genes, we must understand the evolutionary dynamics between the host and the pathogen (McDonald and Linde, 2002) as pathogen isolates do overcome host resistance. Additionally, we need to study about the pathogen avirulence genes to determine the gene mutation it causes in the pathotypes resulting in the fitness loss (Vera Cruz et al., 2000) in the host. To confer durable resistance, the decision about the deployment of race-specific and/or race-non-specific resistance genes should be taken timely in order to maximize the effectiveness of resistance. Another important aspect of successful resistance against pathogen is to understand the mechanism of pathogen attack at different developmental stages (seedling vs. adult) of the host, the infection and colonization process and the host-pathogen interaction which may open up new possibilities for better understanding of the mechanism of downy mildew infection in *Brassica* species.

The ‘R’ genes of plant species mostly contain NBS-LRR domains but apart from classical NBS-LRR types of ‘R’ genes, few other genes encoding receptor like proteins (RLP) responsible for blackleg resistance (*LepR3/Rlm2*) were reported in *B. napus* (Larkan et al., 2015) which recognizes the effectors outside the host cell cytoplasm. This new defense of resistance triggered by apoplastic fungal pathogens was proposed as “effector-triggered defense” (ETD) (Stotz et al., 2014). So, the future studies of resistance genes in *Brassica* species should focus on RLPs and RLKs type resistance genes which may help in the cloning of new ‘R’ genes. In addition to the HR related genes, several other regulatory genes such as transcription factors *WRKY* (Zhou et al., 1997) and calmodulin-binding transcription activator (*CAMTA*) (Rahman et al., 2016) were reported to be involved in defense related mechanism in the pathosystems of *Brassica* (Zhou et al., 1997). The *WRKY* transcription factor gene *BoWRKY6* was found to be responsible for enhancing resistance against downy mildew in transgenic broccoli plants (Jiang et al., 2016). Besides the *WRKY* gene, a pathogenecity-related defensin gene *BoDFN* encoding defense-related cysteine-rich protein was over expressed in *H. parasitica* infected leaves of *Brassica oleracea* var. *italia* improving resistance against downy mildew (Jiang et al., 2012). Fine mapping of the clubroot resistance gene *CRb* and identification of candidate genes revealed the involvement of Rho-binding proteins for clubroot resistance in *B. rapa* (Kato et al., 2013). Likewise, another regulator protein *ZmWAK* was identified conferring resistance to head smut disease in maize (Zuo et al., 2015). All these defense-related regulatory genes need to be characterized in *Brassica* species to decipher their possible role in downy mildew resistance.

The advent of next-generation sequencing technologies has led to the whole genome sequencing of rich genetic resources from different crop species and their wild relatives (reviewed by Bevan et al., 2017). The sequencing of several *Brassica* genomes may allow the understanding of the genetic relationship between various ‘R’ genes discovered between and within different species of *Brassicaceae* family. Till date, the draft genome sequences of almost all the species of *Brassica*; *B. rapa* (Wang et al., 2011), *B. oleracea* (Liu S. et al., 2014; Parkin et al., 2014; Belser et al., 2018), *B. nigra* (Yang et al., 2016), *B. napus* (Chalhoub et al., 2014), *B. juncea* (Yang et al., 2016) have been published. Importantly, the recent publishing of high-quality draft genome of cauliflower (Sun et al., 2019) could serve as a valuable reference to molecular breeding for downy mildew resistance in cauliflower. Additionally, for better access, search, visualization, annotation, structure, and to understand the evolution of the *Brassica* genome, Belser et al. (2018) developed a freely available web-based database^1^, which includes high-quality genome sequences of *B. oleracea* and *B. napus*. The user-friendly database will serve the research community to study the molecular function of genes, evolution of *Brassica* genomes as well as promote molecular breeding research for disease resistance in *B. oleracea*.

As discussed earlier, the triplication of ancestral genomes of *Brassica* has complicated the gene rearrangements leading to the clustering and duplication of ‘R’ genes. So, the reliance on a single reference genome of *Brassica* species may jeopardize ‘R’ gene identification and characterization which may occur as a result of structural variation. Therefore, the variable genes present in other cultivars of the same species need to be searched along with the reference genome for resistance gene identification. Recently, advances were made in pan-genome sequencing representing the diversity of more than one cultivar of the same species. The pan-genome analysis of *B. oleracea* where genomes of nine diverse lines were assembled and compared for structural variation, 81.3% of genes found were core genes and 18.7% were variable genes (Golicz et al., 2016). Importantly, functional analysis of the variable genes suggested the role of the genes and gene families in disease resistance and defense response. The pan-genome approach involving whole-genome gene expression and methylation studies has been used to uncover the structure, function and evolutionary origin of ‘R’ genes (Golicz et al., 2016). So, whole-genome resequencing and pan-genomics studies look promising and could be utilized for identification, characterization and cloning of candidate ‘R’ genes governing downy mildew resistance in *Brassica* species.

Comparative mapping may be used as a tool to study the similarities and differences of resistance genes among closely or distantly related species of *Brassica* to analyze the extent of conservation of ‘synteny’ between genetic maps. All *Brassica* species are closely related to *Arabidopsis thaliana* and structural similarities are found between *B. oleracea* and *Arabidopsis thaliana* genomes. Based on gene homology, the synteny between *Arabidopsis thaliana* and *B. oleracea* genomes has been analyzed (Kowalski et al., 1994; Bohuon et al., 1998; Babula et al., 2003; Lukens et al., 2003; Kaczmarek et al., 2009). Widespread colinearity and conserved genomic sequences between *B. oleracea* and *Arabidopsis thaliana* (Lan et al., 2000; Babula et al., 2003) may assist in the QTL localization in different mapping populations of *B. oleracea*.

Though several QTLs for downy mildew resistance have been identified, most of the causal ‘R’ genes remain unknown till date. The cloning of ‘R’ genes in *Brassica* species may tell us more about the defense mechanism of the plants against the pathogens. Recent advancements in genomics have enabled the researchers to use a pan-transcriptome approach which could reveal the presence and absence of expressed genes in the *Brassica* ‘A’ and ‘C’ genomes (He et al., 2015). A new tool, ‘MutRenSeq’ technology has been used to carry out rapid and precise cloning of ‘R’ genes (Bent, 2016). MutRenSeq technology has been used in cloning the stem rust resistance genes (*Sr22* and *Sr45*) of bread wheat (Steuernagel et al., 2016) and late blight resistance genes (*Rpi*) of potato (Witek et al., 2016). In a complex genome like *Brassica*, methodologies like pan-genome, pan-transcriptome and MutRenSeq technology will lead to the exploitation of novel ‘R’ genes against several important pathogens including downy mildew.

Very recently, genomic selection has been utilized as a powerful approach for crop improvement by identifying minor loci influencing a trait of interest. Genomic selection increases the rate of genetic gain by using the whole genome data to predict the breeding values of the offspring. For durable resistance, the focus of the breeders has now shifted more towards minor quantitative genes rather than single major genes. Different genomic selection models have been successfully captured and genetic variance for disease resistance has been predicted. The recent works demonstrating the application of genomic selection in disease resistance breeding has been reviewed by Poland and Rutkoski (2016) and Bekele et al. (2019). The best studied pathosystem for application of genomic selection models are against different rust pathogens in wheat such as stem rust (Rutkoski et al., 2011, 2014, 2015; Ornella et al., 2012) and yellow/stripe rust (Ornella et al., 2012). In this context, a powerful approach like genomic selection can be implemented in *Brassica* species for disease resistance especially for quantitative resistance.

Functional genomics and metabolomics studies in both the resistant and susceptible cultivars of cauliflower will divulge the key information on host-pathogen interaction. Mining of allelic diversity by EcoTilling or sequence-based allele mining will lead to haplotype identification, diversity analysis of haplotyes, similarity analysis and marker development to differentiate the alleles. Allele mining of genes from wild relatives and land races of cauliflower may detect superior and novel alleles for downy mildew resistance.

## Conclusion

In cauliflower, with the current advances made in the identification of QTLs for downy mildew resistance, there is a long way to go in mapping and cloning of QTLs and its deployment in developing resistant cultivars. With the rapid progress of next-generation sequencing technologies and after publishing of the draft genome sequence of cauliflower and related *Brassica* species, new knowledge of resistance genes/QTLs against important pathogens are becoming available. Furthermore, in near future, several advancements in genomics tools can be expected which will provide new exciting avenues of research in cauliflower and also assist in further improvement of downy mildew resistance. These advancements will bring us closer in developing disease resistant cauliflower and we hope that the limitations can be addressed in the years to come.

## Author Contributions

HG conceived the idea and revised the manuscript. RS wrote the manuscript. HG, ZZ, YS, XS, JW, and HY reviewed and edited the manuscript. All authors have discussed and agreed to the published version of the manuscript.

## Conflict of Interest

The authors declare that the research was conducted in the absence of any commercial or financial relationships that could be construed as a potential conflict of interest.

## Footnotes

1 http://www.genoscope.cns.fr/plants

## References

[B1] Aarts N. Metz M. Holub E. Staskawicz B. J. Daniels M. J. Parker J. E. (1998). Different requirements for EDS1 and NDR1 by disease resistance genes define at least two R-gene-mediated signaling pathways in *Arabidopsis*. *Proc. Natl. Acad. Sci. U.S.A.* 95 10306–10311. 9707643 10.1073/pnas.95.17.10306 PMC21504

[B2] Agnola B. Silue D. Boury S. (2000). Identification of RAPD markers of downy mildew (*Peronospora parasitica*) resistance gene in broccoli (*Brassica oleracea* var. *italica*). *Proc. 3rd Intl. Symp. Brassicas* 64.

[B3] Agrios G. N. (2005). “Control of plant diseases,” in *Plant Pathology*, 5th Edn, ed. Agrios G. N. (London: Elsevier), 295–350.

[B4] Ahmar S. Saeed S. Khan M. H. U. Ullah Khan S. Mora-Poblete F. Kamran M. (2020). A revolution toward gene-editing technology and Its application to crop improvement. *Int. J. Mol. Sci.* 21:5665. 10.3390/ijms21165665 32784649 PMC7461041

[B5] Andersson M. Turesson H. Nicolia A. Falt A. S. Samuelsson M. Hofvander P. (2017). Efficient targeted multiallelic mutagenesis in tetraploid potato (*Solanum tuberosum*) by transient CRISPR-Cas9 expression in protoplasts. *Plant Cell Rep.* 36 117–128. 10.1007/s00299-016-2062-3 27699473 PMC5206254

[B6] Angeles-Shim R. B. Reyes V. P. del Valle M. M. Lapis R. S. Shim J. Sunohara H. (2020). Marker-assisted introgression of quantitative resistance gene pi21 confers broad spectrum resistance to rice blast. *Rice Sci.* 27 113–123. 10.1016/j.rsci.2020.01.002

[B7] Arias T. Beilstein M. A. Tang M. McKain M. R. Pires J. C. (2014). Diversification times among Brassica (*Brassicaceae*) crops suggest hybrid formation after 20 million years of divergence. *Am. J. Bot.* 101 86–91. 10.3732/ajb.1300312 24388963

[B8] Babula D. Kaczmarek M. Barakat A. Delseny M. Quiros C. F. Sadowski J. (2003). Chromosomal mapping of *Brassica oleracea* based on ESTs from *Arabidopsis thaliana*: complexity of the comparative map. *Mol. Genet. Genomics* 268 656–665. 12589440 10.1007/s00438-002-0782-2

[B9] Bains S. S. Sokhi S. S. Jhooty J. S. (1981). Out-break of *Peronospora parasitica* on cauliflower curd in Punjab. *Indian Phytopath.* 34 389–390.

[B10] Barnes W. C. (1968). Development of downy mildew resistant broccoli, cabbage, and collards. *Hort. sci.* 3:78.

[B11] Bekele D. Tesfaye K. Fikre A. (2019). Recent developments in genomic selection for minor gene quantitative disease resistance plant breeding. *J. Plant Pathol. Microbiol.* 10:478. 10.35248/2157-7471.10.478

[B12] Belser C. Istace B. Denis E. Dubarry M. Baurens F.-C. Falentin C. (2018). Chromosome-scale assemblies of plant genomes using nanopore long reads and optical maps. *Nat. Plants* 4 879–887. 10.1038/s41477-018-0289-4 30390080

[B13] Bent A. (2016). Resistance from relatives. *Nat. Biotech.* 2016 620–621. 10.1038/nbt.3591 27281420

[B14] Berg J. A. Hermans F. W. K. Beenders F. Lou L. Vriezen W. H. Visser R. G. F. (2020). Analysis of QTL DM4.1 for downy mildew resistance in cucumber reveals multiple subQTL: a novel RLK as candidate gene for the most important subQTL. *Front. Plant Sci.* 11:569876. 10.3389/fpls.2020.569876 33193500 PMC7649820

[B15] Bevan M. W. Uauy C. Wulff B. B. H. Zhou J. Krasileva K. Clark M. D. (2017). Genomic innovation for crop improvement. *Nature* 543 346–354. 10.1038/nature22011 28300107

[B16] Bittner-Eddy P. Can C. Gunn N. Pinel M. Tör M. Crute I. (1999). Genetic and physical mapping of the RPP13 Locus, in Arabidopsis, responsible for specific recognition of several *Peronospora parasitica* (downy mildew) isolates. *Mol. Plant Microbe Interact.* 12 792–802. 10494631 10.1094/MPMI.1999.12.9.792

[B17] Bittner-Eddy P. Crute I. Holub E. B. Beynon J. (2000). RPP13 is a simple locus in *Arabidopsis thaliana* for alleles that specify downy mildew resistance to different avirulence determinants in *Peronospora parasitica*. *Plant J.* 21 177–188. 10743658 10.1046/j.1365-313x.2000.00664.x

[B18] Bohuon E. J. R. Ramsay L. D. Craft J. A. Arthur A. E. Marshall D. F. Lydiate D. J. (1998). The association of flowering time quantitative trait loci with duplicated regions and candidate loci in *Brassica oleracea*. *Genetics* 150 393–401. 9725855 10.1093/genetics/150.1.393 PMC1460304

[B19] Boller T. Felix G. (2009). A renaissance of elicitors: perception of microbe-associated molecular patterns and danger signals by pattern-recognition receptors. *Annu. Rev. Plant Biol.* 60 379–406. 10.1146/annurev.arplant.57.032905.105346 19400727

[B20] Borrelli V. M. G. Brambilla V. Rogowsky P. Marocco A. Lanubile A. (2018). The ehancement of plant disease resistance using CRISPR/Cas9 technology. *Front. Plant Sci.* 9:1245. 10.3389/fpls.2018.01245 30197654 PMC6117396

[B21] Botella M. A. Parker J. E. Frost L. N. Bittner-Eddy P. D. Beynon J. L. Daniels M. J. (1998). Three genes of the Arabidopsis RPP1 complex resistance locus recognize distinct *Peronospora parasitica avirulence* determinants. *Plant Cell* 10 1847–1860. 10.1105/tpc.10.11.1847 9811793 PMC143951

[B22] Braatz J. Harloff H. J. Mascher M. Stein N. Himmelbach A. Jung C. (2017). CRISPR-Cas9 targeted mutagenesis leads to simultaneous modification of different homoeologous gene copies in polyploid oilseed rape (*Brassica napus*). *Plant Physiol.* 174 935–942. 10.1104/pp.17.00426 28584067 PMC5462057

[B23] Branca F. Bahcevandziev K. Perticone V. Monteiro A. (2005). Sources of resistance to downy mildew (*Peronospora parasitica* (Pers. (ex Fr.) Fr.) in Sicilian germplasm of cauliflower and broccoli. *Biodivers. Conserv.* 14 841–848. 10.1007/s10531-004-0652-9

[B24] Brophy T. F. Laing M. D. (1992). Screening of fungicides for the control of downy mildew on container-grown cabbage seedling. *Crop Prot.* 11 160–164.

[B25] Buckler E. S. Thornsberry J. M. (2002). Plant molecular diversity and applications to genomics. *Curr. Opin. Plant Biol.* 5 107–111. 11856604 10.1016/s1369-5266(02)00238-8

[B26] Cabili M. N. Trapnell C. Goff L. Koziol M. Tazon-Vega B. Regev A. (2011). Integrative annotation of human large intergenic noncoding RNAs reveals global properties and specific subclasses. *Genes Dev.* 25 1915–1927. 10.1101/gad.17446611 21890647 PMC3185964

[B27] Caillaud M. C. Asai S. Rallapalli G. Piquerez S. Fabro G. Jones J. D. (2013). A downy mildew effector attenuates salicylic acid-triggered immunity in Arabidopsis by interacting with the host mediator complex. *PLoS Biol.* 11:e1001732. 10.1371/journal.pbio.1001732 24339748 PMC3858237

[B28] Callaway E. (2018). CRISPR plants now subject to tough GM laws in European Union. *Nature* 560:16. 10.1038/d41586-018-05814-6 30065322

[B29] Cao W. Dong X. Ji J. Yang L. Fang Z. Zhuang M. (2021). BoCER1 is essential for the synthesis of cuticular wax in cabbage (*Brassica oleracea* L. var. *capitata*). *Sci. Hortic.* 277:109801. 10.1016/j.scienta.2020.109801

[B30] Caravalho T. Monteiro A. (1996). Preliminary study on the inheritance of resistance to downy mildew (*Peronospora parasitica* (Pers. Ex. Fr.) at cotyledon stage in Tranchuda cabbage “Algarvia”. *Curciferae News Lett.* 18:104.

[B31] Carlier J. D. Alabac C. A. Coelho P. S. Monteiro A. N. A. Leitão J. M. (2012). The downy mildew resistance locus *Pp523* is located on chromosome C8 of *Brassica oleracea* L. *Plant Breed* 131 170–175.

[B32] Carlier J. D. Alabaça C. S. Sousa N. H. Coelho P. S. Monteiro A. A. Paterson A. H. (2011). Physical mapping in a triplicated genome: mapping the downy mildew resistance locus *Pp523* in *Brassica oleracea* L. *G3 (Bethesda)* 1 593–601. 10.1534/g3.111.001099 22384370 PMC3276173

[B33] Carlsson M. Bothmer R. V. Merker A. (2004). Screening and evaluation of resistance to downy mildew (*Peronospora parasitica*) and clubroot (*Plasmodiophora brassicae*) in genetic resources of *Brassica oleracea*. *Hereditas* 141 293–300. 10.1111/j.1601-5223.2004.01818.x 15703046

[B34] Cavanagh C. Morell M. Mackay I. Powell W. (2008). From mutations to MAGIC: resources for gene discovery, validation and delivery in crop plants. *Curr. Opin. Plant Biol.* 11 215–221. 10.1016/j.pbi.2008.01.002 18295532

[B35] Cavell A. C. Lydiate D. J. Parkin I. A. P. Dean C. Trick M. (1998). Collinearity between a 30-centimorgan segment of *Arabidopsis thaliana* chromosome 4 and duplicated regions within the *Brassica napus* genome. *Genome* 41 62–69. 9549059

[B36] Chalhoub B. Denoeud F. Liu S. Parkin I. A. Tang H. Wang X. (2014). Early allopolyploid evolution in the post-Neolithic *Brassica napus* oilseed genome. *Science* 345 950–953. 10.1126/science.1253435 25146293

[B37] Chang T. Guan M. Zhou B. Peng Z. Xing M. Wang X. (2021). Progress of CRISPR/Cas9 technology in breeding of *Brassica napus*. *Oil Crop Sci.* 6 53–57. 10.1016/j.ocsci.2021.03.005

[B38] Chatterjee S. S. (1993). “Cole crops,” in *Vegetable Crops*, eds Bose T. K. Som M. G. Abir J. (India: Naya Prokash), 125–223.

[B39] Chen X. F. Qing-Hua M. A. Jin-Hua M. U. Wang B. C. (2015). PRs genes expression in Chinese cabbage induction by *Peronospora parasitica*. *J. Jilin Agric. Sci.* 40 60–64.

[B40] Choi Y. J. Hong S. B. Shin H. D. (2003). Diversity of the *Hyaloperonospora parasitica* complex from core brassicaceous hosts based on ITS rDNA sequences. *Mycol. Res.* 107 1314–1322. 10.1017/S0953756203008578 15000233

[B41] Chukwu S. C. Rafii M. Y. Ramlee S. I. Ismail S. I. Hasan M. M. Oladosu Y. A. (2019). Bacterial leaf blight resistance in rice: a review of conventional breeding to molecular approach. *Mol. Biol. Rep.* 46 1519–1532. 10.1007/s11033-019-04584-2 30628024

[B42] Coelho P. Leckie D. Bahcevandziev K. Valério L. Astley D. Boukema I. (1998). The relationship between cotyledon and adult plant resistance to downy mildew (*Peronospora parasitica*) in *Brassica oleracea*. *Proc 2nd Int Symp Brassicas. Acta Hort* 459 335–342.

[B43] Coelho P. S. Monteiro A. A. (2003a). Expression of resistance to downy mildew at cotyledon and adult plant stages in *Brassica oleracea*. *Euphytica* 133 279–284.

[B44] Coelho P. S. Monteiro A. A. (2003b). Inheritance of downy mildew resistance in mature broccoli plants. *Euphytica* 131 65–69.

[B45] Coelho P. S. Vicente J. G. Monteiro A. A. Holub E. B. (2012). Pathotypic diversity of *Hyaloperonospora brassicae* collected from *Brassica oleracea*. *Eur. J. Plant Pathol.* 134 763–771. 10.1007/s10658-012-0052-z

[B46] Coker T. L. Cevik V. Beynon J. L. Gifford M. L. (2015). Spatial dissection of the *Arabidopsis thaliana* transcriptional response to downy mildew using fluorescence activated cell sorting. *Front. Plant Sci.* 6:527. 10.3389/fpls.2015.00527 26217372 PMC4498041

[B47] Cooley M. B. Pathirana S. Hj W. Kachroo P. Klessig D. F. (2000). Members of the *Arabidopsis* HRT/RPP8 family of resistance genes confer resistance to both viral and oomycete pathogens. *Plant Cell* 12 663–676. 10810142 10.1105/tpc.12.5.663 PMC139919

[B48] Crute I. R. Gordon P. L. (1987). Downy mildew. *Rep. Natn. Veg. Res. Sta.* 54.

[B49] Cui J. Jiang N. Meng J. Yang G. Liu W. Zhou X. (2019). LncRNA33732-respiratory burst oxidase module associated with WRKY1 in tomato–*Phytophthora infestans* interactions. *Plant J.* 97 933–946. 10.1111/tpj.14173 30472748

[B50] Cui J. Luan Y. Jiang N. Bao H. Meng J. (2017). Comparative transcriptome analysis between resistant and susceptible tomato allows the identification of lncRNA16397 conferring resistance to *Phytophthora infestans* by co-expressing glutaredoxin. *Plant J.* 89 577–589. 10.1111/tpj.13408 27801966

[B51] Das G. Rao G. J. (2015). Molecular marker assisted gene stacking for biotic and abiotic stress resistance genes in an elite rice cultivar. *Front. Plant Sci.* 6:698. 10.3389/fpls.2015.00698 26483798 PMC4588116

[B52] Datta K. Baisakh N. Maung Thet K. Tu J. Datta S. (2002). Pyramiding transgenes for multiple resistance in rice against bacterial blight, yellow stem borer and sheath blight. *Theor. Appl. Genet.* 106 1–8. 10.1007/s00122-002-1014-1 12582865

[B53] de Jonge R. van Esse H. P. Maruthachalam K. Bolton M. D. Santhanam P. Saber M. K. (2012). Tomato immune receptor Ve1 recognizes effector of multiple fungal pathogens uncovered by genome and RNA sequencing. *Proc. Natl. Acad. Sci. U.S.A.* 109 5110–5115. 10.1073/pnas.1119623109 22416119 PMC3323992

[B54] de Toledo Thomazella D. Brail Q. Dahlbeck D. Staskawicz B. (2016). CRISPR-Cas9 mediated mutagenesis of a DMR6 ortholog in tomato confers broad-spectrum disease resistance. *BioRxiv [Preprint]* 10.1101/064824

[B55] Dickson M. H. Petzoldt R. (1993). Plant age and isolate source affect expression of downy mildew resistance in broccoli. *Hort. Sci.* 28 730–731.

[B56] Dong O. X. Ronald P. C. (2019). Genetic engineering for disease resistance in plants: Recent progress and future perspectives. *Plant Physiol.* 180 26–38. 10.1104/pp.18.01224 30867331 PMC6501101

[B57] Dormatey R. Sun C. Ali K. Coulter J. A. Bi Z. Bai J. (2020). Gene pyramiding for sustainable crop improvement against biotic and abiotic stresses. *Agronomy* 10:1255. 10.3390/agronomy10091255

[B58] Eulgem T. Tsuchiya T. Wang X. J. Beasley B. Cuzick A. Tor M. (2007). EDM2 is required for RPP7-dependent disease resistance in *Arabidopsis* and affects RPP7 transcript levels. *Plant J.* 49 829–839. 17253987 10.1111/j.1365-313X.2006.02999.x

[B59] Eulgem T. Weigman V. J. Chang H. S. McDowell J. M. Holub E. B. Glazebrook J. (2004). Gene expression signatures from three genetically separable resistance gene signaling pathways for downy mildew resistance. *Plant Physiol.* 135 1129–1144. 10.1104/pp.104.040444 15181204 PMC514145

[B60] FAOSTAT. (2018). *Food and Agriculture Organization of the United Nations.* Available online at: http://faostat.fao.org (accessed December 15, 2020).

[B61] Farinho M. J. Coelho P. S. Monteiro A. A. Leitao J. M. (2000). RAPD and AFLP markers linked to *Peronospora parasitica* resistance genes in broccoli. *Proc. 3rd Intl. Symp. Brassicas* 75.

[B62] Farinhó M. Coelho P. Carlier J. Svetleva D. Monteiro A. Leitão J. (2004). Mapping of a locus for adult plant resistance to downy mildew in broccoli (*Brassica oleracea* con var. *italica*). *Theor. Appl. Genet* 109 1392–1398. 15278283 10.1007/s00122-004-1747-0

[B63] Farinhó M. Coelho P. Monteiro A. Leitão J. (2007). SCAR and CAPS markers flanking the *Brassica oleracea* L. *Pp523* downy mildew resistance locus demarcate a genomic region syntenic to the top arm end of *Arabidopsis thaliana* L. chromosome 1. *Euphytica* 157 215–221.

[B64] Farnham M. W. Wang M. Thomas C. E. (2002). A single dominant gene for downy mildew resistance in broccoli. *Euphytica* 128 405–407.

[B65] Gaikwad A. P. Kakade D. S. Nimbalkar C. A. Desai U. T. (2004). Control of downy mildew (*Peronospora parasitica*) of cauliflower (*Brassica oleracea* L. var. *botrytis*) in nursery. *Indian J. Agric. Sci.* 74 230–232.

[B66] Gao C. Sun J. Dong Y. Wang C. Xiao S. Mo L. (2020). Comparative transcriptome analysis uncovers regulatory roles of long non-coding RNAs involved in resistance to powdery mildew in melon. *BMC Genomics* 21:125. 10.1186/s12864-020-6546-8 32024461 PMC7003419

[B67] Gao M. Li G. Yang B. Farnham M. Quiros C. (2003). “Search for candidate genes for downy mildew resistance in broccoli,” in *Proceedings of the Plant and Animal Genomes XI Conf. Abstract No 476*, San Diego, CA.

[B68] Gao M. Li G. Yang B. Qiu D. Farnham M. Quiros C. (2007). High-density *Brassica oleracea* linkage map: identification of useful new linkages. *Theor. Appl. Genet.* 115 277–287. 10.1007/s00122-007-0568-3 17592603

[B69] Gao R. Liu P. Irwanto N. Loh R. Wong S. M. (2016). Upregulation of LINC-AP2 is negatively correlated with AP2 gene expression with Turnip crinkle virus infection in *Arabidopsis thaliana*. *Plant Cell Rep.* 35 2257–2267. 10.1007/s00299-016-2032-9 27473526

[B70] Gao T. Yu S. Zhang F. Chen X. Yu Y. Zhang D. (2014). Expression analysis of major genes involved in signaling pathways during infection of Chinese cabbage with *Hyaloperonospora brassicae*. *Sci. Hortic.* 167 27–35. 10.1016/j.scienta.2013.12.020

[B71] Ge X. T. Li H. Han S. Sivasithamparam K. Barbetti M. J. (2008). Evaluation of Australian *Brassica napus* genotypes for resistance to the downy mildew pathogen, *Hyaloperonospora parasitica*. *Aust. J. Agric. Res.* 59 1030–1034. 10.1071/AR08032

[B72] Giovannelli J. L. Farnham M. W. Wang M. (2002). Development of sequence characterized amplified region markers linked to downy mildew resistance in broccoli. *J. Am. Soc. Hortic. Sci.* 127 597–601.

[B73] Glazebrook J. Zook M. Mert F. Kagan I. Rogers E. R. Crute I. R. (1997). Phytoalexin deficient mutants of Arabidopsis reveal that PAD4 encodes a regulatory factor and that four PAD genes contribute to downy mildew resistance. *Genetics* 146 381–392. 9136026 10.1093/genetics/146.1.381 PMC1207952

[B74] Golicz A. A. Batley J. Edwards D. (2016). Towards plant pangenomics. *Plant Biotechnol. J.* 14 1099–1105. 26593040 10.1111/pbi.12499 PMC11388911

[B75] Goodwin S. B. Allard R. W. Webster R. K. (1990). A nomenclature for *Rhynchosporium secalis* pathotypes. *Phytopathology* 80 1330–1336. 10.1094/Phyto-80-1330

[B76] Gunn N. Byrne J. Holub E. B. (2002). “Outcrossing of two homothallic isolates of *Peronospora parasitica* and segregation of avirulence matching six resistance loci in *Arabidopsis thaliana*,” in *Advances in Downy Mildew research*, eds Spencer-Phillips P. T. N. Gisi U. Lebeda A. (Dordrecht: Kluwer Academic Publishers), 185–188.

[B77] He Z. Cheng F. Li Y. Wang X. Parkin I. A. P. Chalhoub B. (2015). Construction of Brassica A and C genome-based ordered pan-transcriptomes for use in rapeseed genomic research. *Data Brief.* 4 357–362. 10.1016/j.dib.2015.06.016 26217816 PMC4510581

[B78] Holub E. B. (1997). “Organization of resistance genes in *Arabidopsis*,” in *The Gene-for-Gene Relationship in Plant Parasite Interactions*, eds Crute I. R. Holub E. B. Burdon J. J. (Wallingford: CAB International), 5–26.

[B79] Hoser-Krauze J. Lakowaska-Ryk E. Antosik J. (1995). The inheritance of resistance of some *Brassica oleracea* L. cultivars and lines to downy mildew (*P. parasitica*). *Ex. Fr. J. Appl. Genet.* 36 27–33.

[B80] Hoser-Krauze J. Lakowska-Ryk E. Antosik J. (1984). Resistance of cauliflower and broccoli (*Brassica oleracea* L. var. *botrytis* L.), seedlings to downy mildew (*Perenospora parasitica*). *Eucarpia Cruciferae Newl.* 9:92.

[B81] Hou X. Cui J. Liu W. Jiang N. Zhou X. Qi H. (2020). LncRNA39026 enhances tomato resistance to *Phytophthora infestans* by decoying miR168a and inducing PR gene expression. *Phytopathology* 110 873–880. 10.1094/PHYTO-12-19-0445-R 31876247

[B82] Innark P. Panyanitikoon H. Khanobdee C. Samipak S. Jantasuriyarat C. (2020). QTL identification for downy mildew resistance in cucumber using genetic linkage map based on SSR markers. *J. Genet.* 99:81. 10.1007/s12041-020-01242-6 33361633

[B83] Jamaloddin M. Durga Rani C. V. Swathi G. Anuradha C. Vanisri S. Rajan C. P. D. (2020). Marker Assisted Gene Pyramiding (MAGP) for bacterial blight and blast resistance into mega rice variety “Tellahamsa”. *PLoS One.* 15:e0234088. 10.1371/journal.pone.0234088 32559183 PMC7304612

[B84] Jensen B. D. Hockenhull J. Munk L. (1999a). Seedling and adult plant resistance to downy mildew in cauliflower (*Brassica oleracea* var. *botrytis*). *Plant Pathol.* 48 604–612.

[B85] Jensen B. D. Værbak S. Munk L. Andersen S. B. (1999b). Characterization and inheritance of partial resistance to downy mildew, *Peronospora parasitica*, in breeding material of broccoli, *Brassica oleracea* convar. *botrytis* var. *italica*. *Plant Breed.* 118 549–554. 10.1046/j.1439-0523.1999.00409.x

[B86] Ji X. Zhang H. Zhang Y. Wang Y. Gao C. (2015). Establishing a CRISPR-Cas-like immune system conferring DNA virus resistance in plants. *Nat. Plants* 1:15144. 10.1038/nplants.2015.144 27251395

[B87] Jiang L. Chen Y. Luo L. Peck S. C. (2018). Central roles and regulatory mechanisms of dual-specificity MAPK phosphatases in developmental and stress signaling. *Front. Plant Sci.* 9:1697. 10.3389/fpls.2018.01697 30515185 PMC6255987

[B88] Jiang M. He C. M. Miao L. X. Zhang Y. C. (2012). Overexpression of a broccoli defensin gene BoDFN enhances downy mildew resistance. *J. Integr. Agric.* 11 1137–1144. 10.1016/S2095-3119(12)60107-5

[B89] Jiang M. Liu Q. E. Liu Z. N. Li J. Z. He C. M. (2016). Over-expression of a WRKY transcription factor gene BoWRKY6 enhances resistance to downy mildew in transgenic broccoli plants. *Aus. Plant Pathol.* 45 327–334. 10.1007/s13313-016-0416-5

[B90] Joos H. J. Mauch-Mani B. Slusarenko A. J. (1996). Molecular mapping of the *Arabidopsis* locus RPP11 which conditions isolate-specific hypersensitive resistance against downy mildew in ecotype RLD. *Theor. Appl. Genet.* 92 281–284. 24166179 10.1007/BF00223387

[B91] Joseph M. Gopalakrishnan S. Sharma R. K. Singh V. P. Singh A. K. Singh N. K. (2004). Combining bacterial blight resistance and Basmati quality characteristics by phenotypic and molecular marker-assisted selection in rice. *Mol. Breed.* 13 377–387.

[B92] Kaczmarek M. Koczyk G. Ziolkowski P. A. Babula-Skowronska D. Sadowski J. (2009). Comparative analysis of the *Brassica oleracea* genetic map and the *Arabidopsis thaliana* genome. *Genome* 52 620–633. 10.1139/G09-035 19767893

[B93] Kato T. Hatakayema K. Fukino N. Matsumoto S. (2013). Fine mapping of the clubroot resistance gene CRb and development of a useful selectable marker in *Brassica rapa*. *Breed. Sci.* 63 116–124. 10.1270/jsbbs.63.116 23641188 PMC3621437

[B94] Kim S. Song Y. H. Lee J. Y. Choi S. R. Dhandapani V. Jang C. S. (2011). Identification of the BrRHP1 locus that confers resistance to downy mildew in Chinese cabbage (*Brassica rapa* ssp. *pekinensis*) and development of linked molecular markers. *Theor Appl Genet.* 123 1183–1192. 10.1007/s00122-011-1658-9 21814857

[B95] Kontaxis D. G. Mayberry K. S. Rubatzky V. E. (1979). Reaction of cauliflower cultivars to downy mildew in Imperial Valley. *California Agric.* 33:19.

[B96] Kowalski S. P. Lan T. H. Feldmann K. A. Paterson A. H. (1994). Comparative mapping of *Arabidopsis thaliana* and *Brassica oleracea* chromosomes reveals islands of conserved organization. *Genetics* 138 499–510. 7828831 10.1093/genetics/138.2.499 PMC1206166

[B97] Kumar V. Jain M. (2015). The CRISPR-Cas system for plant genome editing: advances and opportunities. *J. Exp. Bot.* 66 47–57. 10.1093/jxb/eru429 25371501

[B98] Lagercrantz U. (1998). Comparative mapping between *Arabidopsis thaliana* and *Brassica nigra* indicates that Brassica genomes have evolved through extensive genome replication accompanied by chromosome fusions and frequent rearrangements. *Genetics* 150 1217–1228. 9799273 10.1093/genetics/150.3.1217 PMC1460378

[B99] Laloi G. Vergne E. Durel C. E. Le Cam B. Caffier V. (2017). Efficiency of pyramiding of three quantitative resistance loci to apple scab. *Plant Pathol.* 66 412–422. 10.1111/ppa.12581

[B100] Lan T. H. DelMonte T. A. Reischmann K. P. Hyman J. Kowalski S. P. McFerson J. (2000). An EST-enriched comparative map of *Brassica oleracea* and *Arabidopsis thaliana*. *Genome Res.* 10 776–788. 10.1101/gr.10.6.776 10854410 PMC310908

[B101] Langner T. Kamoun S. Belhaj K. (2018). CRISPR Crops: plant genome editing toward disease resistance. *Annu. Rev. Phytopathol.* 56 479–512. 10.1146/annurev-phyto-080417-050158 29975607

[B102] Larkan N. J. Ma L. Borhan M. H. (2015). The *Brassica napus* receptorlike protein RLM2 is encoded by a second allele of the LepR3/Rlm2 blackleg resistance locus. *Plant Biotechnol. J.* 13 983–992. 10.1111/pbi.12341 25644479

[B103] Lawrenson T. Shorinola O. Stacey N. Li C. Østergaard L. Patron N. (2015). Induction of targeted, heritable mutations in barley and *Brassica oleracea* using RNA-guided Cas9 nuclease. *Genome Biol.* 16:258. 10.1186/s13059-015-0826-7 26616834 PMC4663725

[B104] Li H. Yu S. Zhang F. Yu Y. Zhao X. Zhang D. (2011). Development of molecular markers linked to the resistant QTL for downy mildew in *Brassica rapa* L. ssp. *pekinensis*. *Hereditas (Beijing)* 33 1271–1278. 10.3724/SP.J.1005.2011.01271 22120085

[B105] Li J. Ding Q. Wang F. Li H. Zhang Y. Liu L. (2018). Genome-wide gene expression profiles in response to downy mildew in Chinese cabbage (*Brassica rapa* L. ssp. *pekinensis*). *Eur. J. Plant Pathol.* 151 861–873. 10.1007/s10658-018-1427-6

[B106] Liu S. Liu Y. Yang X. Tong C. Edwards D. Parkin I. A. (2014). The *Brassica oleracea* genome reveals the asymmetrical evolution of polyploid genomes. *Nat. Commun.* 5:3930. 10.1038/ncomms4930 24852848 PMC4279128

[B107] Liu W. Liu J. Triplett L. Leach J. E. Wang G. L. (2014). Novel insights into rice innate immunity against bacterial and fungal pathogens. *Annu. Rev. Phytopathol.* 52 213–241. 10.1146/annurev-phyto-102313-045926 24906128

[B108] Liu Y. Chen L. Liu Y. Dai H. He J. Kang H. (2016). Marker assisted pyramiding of two brown planthopper resistance genes, Bph3 and Bph27 (t), into elite rice Cultivars. *Rice (N Y)* 9:27. 10.1186/s12284-016-0096-3 27246014 PMC4887400

[B109] Lukens L. Zou F. Lydiate D. Parkin I. Osborn T. (2003). Comparison of a *Brassica oleracea* genetic map with the genome of *Arabidopsis thaliana*. *Genetics* 164 359–372. 12750346 10.1093/genetics/164.1.359 PMC1462567

[B110] Ma C. Liu M. Li Q. Si J. Ren X. Song H. (2019). Efficient BoPDS gene editing in cabbage by the CRISPR/Cas9 System. *Hortic. Plant J.* 5 164–169. 10.1016/j.hpj.2019.04.001

[B111] Mahajan V. Gill H. S. More T. A. (1995). Inheritance of downy mildew resistance in Indian cauliflower (group III). *Euphytica* 86 1–3.

[B112] Mahajan V. Gill H. S. Singh R. (1991). Screening of cauliflower germplasm lines against downy mildew. *Cruciferae Newl.* 14/15 148–149.

[B113] Manzanares-Dauleux M. Delourme R. Baron F. Thomas G. (2000). Mapping of one major gene and of QTLs involved in resistance to clubroot in *Brassica napus*. *Theor. Appl. Genet.* 101 885–891. 10.1007/s001220051557

[B114] Matsumoto E. Ueno H. Aruga D. Sakamoto K. Hayashida N. (2012). Accumulation of three clubroot resistance genes through marker-assisted selection in Chinese cabbage (*Brassica rapa* ssp. *pekinensis*). *J. Jpn. Soc. Hort. Sci.* 81 184–190. 10.2503/jjshs1.81.184

[B115] McDonald B. A. Linde C. (2002). Pathogen population genetics, evolutionary potential, and durable resistance. *Annu. Rev. Phytopathol.* 40 349–379. 10.1146/annurev.phyto.40.120501.101443 12147764

[B116] McDowell J. Cuzick A. Can C. Beynon J. Dangl J. L. Holub E. B. (2000). Downy mildew (*Peronospora parasitica*) resistance genes in *Arabidopsis* vary in functional requirements for NDR1, EDS1, NPR1 and salicylic acid accumulation. *Plant J.* 22 523–529. 10886772 10.1046/j.1365-313x.2000.00771.x

[B117] McDowell J. M. Dhandaydham M. Long T. A. Aarts M. G. Goff S. Holub E. B. (1998). Intragenic recombination and diversifying selection contribute to the evolution of downy mildew resistance at the RPP8 locus of Arabidopsis. *Plant Cell* 10 1861–1874. 10.1105/tpc.10.11.1861 9811794 PMC143965

[B118] McDowell J. M. Williams S. G. Funderburg N. T. Eulgem T. Dangl J. L. (2005). Genetic analysis of developmentally regulated resistance to downy mildew (*Hyaloperonospora parasitica*) in *Arabidopsis thaliana*. *Mol. Plant Microbe Interact.* 18 1226–1234. 10.1094/MPMI-18-1226 16353557

[B119] Miladinovic D. Antunes D. Yildirim K. Bakhsh A. Cvejić S. Kondić-Špika A. (2021). Targeted plant improvement through genome editing: from laboratory to field. *Plant Cell Rep.* 40 935–951. 10.1007/s00299-020-02655-4 33475781 PMC8184711

[B120] Mitchell-Olds T. Bradley D. (1996). Genetics of Brassica rapa 3. Costs of disease resistance to three fungal pathogens. *Evolution* 50 1859–1865. 28565608 10.1111/j.1558-5646.1996.tb03572.x

[B121] Mohammed A. E. You M. P. Banga S. S. Barbetti M. J. (2018). Resistances to downy mildew (*Hyaloperonospora brassicae*) in diverse Brassicaceae offer new disease management opportunities for oilseed and vegetable crucifer industries. *Eur. J. Plant Pathol.* 153 915–929. 10.1007/s10658-018-01609-7

[B122] Monteirio A. A. Coelho P. S. Bahcevandziev K. Valerio L. (2005). Inheritance of downy mildew resistance at cotyledon and adult-plant stages in ‘Couve Algarvia’ (*Brassica oleracea* var. *tronchuda*). *Euphytica* 141 85–92.

[B123] Monteirio A. A. Williams P. H. (1989). The exploration of genetic resources of Portuguese cabbage and kale for resistance to several Brassica diseases. *Euphytica* 41 215–225.

[B124] Mushtaq M. Sakina A. Wani S. H. Shikari A. B. Tripathi P. Zaid A. (2019). Harnessing genome editing techniques to engineer disease resistance in plants. *Front. Plant Sci.* 10:550. 10.3389/fpls.2019.00550 31134108 PMC6514154

[B125] Nagaharu U. (1935). Genome analysis in Brassica with special reference to the experimental formation of *Brassica napus* and peculiar mode of fertilization. *Japan J. Bot.* 7 389–452.

[B126] Narayanan N. N. Baisakh N. Vera Cruz C. M. Gnanamanickam S. S. Datta K. Datta S. K. (2002). Molecular breeding for the development of blast and bacterial blight resistance in rice cv. IR50. *Crop Sci.* 42 2072–2079.

[B127] Nascimento-Gavioli M. C. Agapito-Tenfen S. Z. Nodari R. O. Welter L. J. Sanchez Mora F. D. Saifert L. (2017). Proteome of *Plasmopara viticola*-infected Vitis vinifera provides insights into grapevine Rpv1/Rpv3 pyramided resistance to downy mildew. *J. Proteom.* 151 264–274. 10.1016/j.jprot.2016.05.024 27235723

[B128] Natti J. J. (1958). Resistance of broccoli and other crucifers to downy mildew. *Plant Dis. Rptr.* 42 656–662.

[B129] Natti J. J. Atkin J. D. (1960). Inheritance of downy mildew resistance in broccoli. *Phytopatholgy* 50:241.

[B130] Natti J. J. Dickson M. H. Atkin J. D. (1967). Resistance of *Brassica oleracea* varieties to downy mildew. *Phytopathology* 1967 144–147.

[B131] Nekrasov V. Wang C. Win J. Lanz C. Weigel D. Kamoun S. (2017). Rapid generation of a transgene-free powdery mildew resistant tomato by genome deletion. *Sci Rep.* 7:482. 10.1038/s41598-017-00578-x 28352080 PMC5428673

[B132] Niu X. K. Leung H. Williams P. H. (1983). Sources and nature of resistance to downy mildew and turnip mosaic in Chinese cabbage. *J. American Soc. Hort. Sci.* 5 775–778.

[B133] Okuzaki A. Ogawa T. Koizuka C. Kaneko K. Inaba M. Imamura J. (2018). CRISPR/Cas9-mediated genome editing of the fatty acid desaturase 2 gene in *Brassica napus*. *Plant Physiol. Biochem.* 131 63–69. 10.1016/j.plaphy.2018.04.025 29753601

[B134] Oliva R. Ji C. Atienza-Grande G. Huguet-Tapia J. C. Perez-Quintero A. Li T. (2019). Broad-spectrum resistance to bacterial blight in rice using genome editing. *Nat. Biotechno.* 37 1344–1350. 10.1038/s41587-019-0267-z 31659337 PMC6831514

[B135] O’Neill C. Bancroft I. (2000). Comparative physical mapping of segments of the genome of *Brassica oleracea* var. *alboglabra* that are homoeologous to sequenced regions of chromosomes 4 and 5 of *Arabidopsis thaliana*. *Plant J.* 23 233–243. 10929117 10.1046/j.1365-313x.2000.00781.x

[B136] Ornella L. Singh S. Perez P. Burgueno J. Singh R. Tapia E. (2012). Genomic prediction of genetic values for resistance to wheat rusts. *Plant Genome* 5 136–148. 10.3835/plantgenome2012.07.0017

[B137] Pandey K. K. Pandey P. K. Singh B. Kalloo G. Kapoor K. S. (2001). Sources of resistance to downy mildew disease in Asiatic group of cauliflower. *Veg. Sci.* 28 55–57.

[B138] Parker J. E. Coleman M. J. Szabò V. Frost L. N. Schmidt R. Van Der Biezen E. A. (1997). The Arabidopsis downy mildew resistance gene *RPP5* shares similarity to the toll and interleukin-1 receptors with N and L6. *Plant Cell* 9 879–894. 10.1105/tpc.9.6.879 9212464 PMC156965

[B139] Parker J. E. Holub E. B. Frost L. N. Falk A. Gunn N. D. Daniels M. J. (1996). Characterization of eds1, a mutation in Arabidopsis suppressing resistance to *Peronospora parasitica* specified by several different RPP genes. *Plant Cell* 8 2033–2046. 8953768 10.1105/tpc.8.11.2033 PMC161332

[B140] Parker J. E. Szabo V. Staskawicz B. J. Lister B. J. Dean C. Daniels M. J. (1993). Phenotypic characterization and molecular mapping of the *Arabidopsis thaliana* locus, *RPP5*, determining disease resistance to *Peronospora parasitica*. *Plant J.* 4 821–831.

[B141] Parkin I. A. Gulden S. M. Sharpe A. G. Lukens L. Trick M. Osborn T. C. (2005). Segmental structure of the *Brassica napus* genome based on comparative analysis with *Arabidopsis thaliana*. *Genetics* 171 765–781. 10.1534/genetics.105.042093 16020789 PMC1456786

[B142] Parkin I. A. P. Koh C. Tang H. Robinson S. J. Kagale S. Clarke W. E. (2014). Transcriptome and methylome profiling reveals relics of genome dominance in the mesopolyploid *Brassica oleracea*. *Genome Biol.* 15:R77. 10.1186/gb-2014-15-6-r77 24916971 PMC4097860

[B143] Peng A. Chen S. Lei T. Xu L. He Y. Wu L. (2017). Engineering canker-resistant plants through CRISPR/Cas9-targeted editing of the susceptibility gene CsLOB1 promoter in citrus. *Plant Biotechnol. J.* 15 1509–1519. 10.1111/pbi.12733 28371200 PMC5698050

[B144] Plissonneau C. Rouxel T. Chèvre A. M. Van DeWouw A. P. Balesdent M. H. (2017). One gene-one name: the AvrLmJ1 avirulence gene of *Leptosphaeria maculans* is AvrLm5. *Mol. Plant Pathol.* 19 1012–1016. 10.1111/mpp.12574 28661570 PMC6638039

[B145] Poland J. Rutkoski J. (2016). Advances and challenges in genomic selection for disease resistance. *Annu. Rev. Phytopathol.* 54 79–98. 27491433 10.1146/annurev-phyto-080615-100056

[B146] Qi L. Ma G. (2019). Marker-assisted gene pyramiding and the reliability of using SNP markers located in the recombination suppressed regions of sunflower (*Helianthus annuus* L.). *Genes (Basel)* 11:10. 10.3390/genes11010010 31861950 PMC7016752

[B147] Qi L. L. Talukder Z. I. Hulke B. S. Foley M. E. (2017). Development and dissection of diagnostic SNP markers for the downy mildew resistance genes Pl Arg and Pl 8 and maker-assisted gene pyramiding in sunflower (*Helianthus annuus* L.). *Mol. Genet. Genomics* 292 551–563. 10.1007/s00438-017-1290-8 28160079

[B148] Rahman H. Xu Y. P. Zhang X. R. Cai X. Z. (2016). *Brassica napus* genome possesses extraordinary high number of CAMTA Genes and CAMTA3 contributes to PAMP triggered immunity and resistance to *Sclerotinia sclerotiorum*. *Front. Plant Sci.* 7:581. 10.3389/fpls.2016.00581 27200054 PMC4854897

[B149] Ramalingam J. Raveendra C. Savitha P. Vidya V. Chaithra T. L. Velprabakaran S. (2020). Gene pyramiding for achieving enhanced resistance to bacterial blight, blast, and bheath blight diseases in rice. *Front. Plant Sci.* 11:591457. 10.3389/fpls.2020.591457 33329656 PMC7711134

[B150] Rocherieux J. Glory P. Giboulot A. Boury S. Barbeyron G. Thomas G. (2004). Isolate-specific and broad-spectrum QTLs are involved in the control of clubroot in *Brassica oleracea*. *Theor. Appl. Genet.* 108 1555–1563. 10.1007/s00122-003-1580-x 15007504

[B151] Rutkoski J. Singh R. P. Huerta-Espino J. Bhavani S. Poland J. (2015). Genetic gain from phenotypic and genomic selection for quantitative resistance to stem rust of wheat. *Plant Genome* 8 1–10. 10.3835/plantgenome2014.10.0074 33228306

[B152] Rutkoski J. E. Heffner E. L. Sorrells M. E. (2011). Genomic selection for durable stem rust resistance in wheat. *Euphytica* 179 161–173. 10.1007/s10681-010-0301-1

[B153] Rutkoski J. E. Poland J. A. Singh R. P. Huerta-Espino J. Bhavani S. (2014). Genomic selection for quantitative adult plant stem rust resistance in wheat. *Plant Genome* 7 1–10. 10.3835/plantgenome2014.02.0006

[B154] Saha P. Ghoshal C. Ray S. Saha N. D. Srivastava M. Kalia P. (2020). Genetic analysis of downy mildew resistance and identification of molecular markers linked to resistance gene Ppa207 on chromosome 2 in cauliflower. *Euphytica* 216:183. 10.1007/s10681-020-02696-6

[B155] Saifert L. Sánchez-Mora F. D. Assumpção W. T. Zanghelini J. A. Giacometti R. Novak E. I. (2018). Marker-assisted pyramiding of resistance loci to grape downy mildew. *Pesqui. Agropecu. Bras.* 53 602–610. 10.1590/s0100-204x2018000500009

[B156] Salava H. Thula S. Mohan V. Kumar R. Maghuly F. (2021). Application of genome editing in tomato breeding: Mechanisms, Advances, and Prospects. *Int. J. Mol. Sci.* 22:682. 10.3390/ijms22020682 33445555 PMC7827871

[B157] Samsampour D. Maleki Zanjani B. Pallavi J. K. Singh A. Charpe A. Gupta S. K. (2010). Identification of molecular markers linked to adult plant leaf rust resistance gene Lr48 in wheat and detection of Lr48 in the Thatcher near-isogenic line with gene Lr25. *Euphytica* 174 337–342. 10.1007/s10681-009-0114-2

[B158] Sanchez-Martin J. Keller B. (2019). Contribution of recent technological advances to future resistance breeding. *Theor. Appl. Genet.* 132 713–732. 10.1007/s00122-019-03297-1 30756126

[B159] Schenke D. Cai D. (2020). Applications of CRISPR/Cas to improve crop disease resistance: beyond inactivation of susceptibility factors. *iScience* 23:101478. 10.1016/j.isci.2020.101478 32891884 PMC7479627

[B160] Schmidt S. M. Belisle M. Frommer W. B. (2020). The evolving landscape around genome editing in agriculture: Many countries have exempted or move to exempt forms of genome editing from GMO regulation of crop plants. *EMBO Rep.* 21:e50680. 10.15252/embr.202050680 32431018 PMC7271327

[B161] Schwander F. Eibach R. Fechter I. Hausmann L. Zyprian E. Topfer R. (2012). Rpv10: a new locus from the Asian Vitis gene pool for pyramiding downy mildew resistance loci in grapevine. *Theor. Appl. Genet.* 124 163–176. 10.1007/s00122-011-1695-4 21935694

[B162] Servin B. Martin O. C. Mezard M. Hospital F. (2004). Toward a theory of marker-assisted gene pyramiding. *Genetics* 168 513–523. 10.1534/genetics.103.023358 15454561 PMC1448128

[B163] Shah N. Sun J. Yu S. Yang Z. Wang Z. Huang F. (2019). Genetic variation analysis of field isolates of clubroot and their responses to *Brassica napus* lines containing resistant genes *CRb* and *PbBa8.1* and their combination in homozygous and heterozygous state. *Mol. Breed* 39:153. 10.1007/s11032-019-1075-3

[B164] Sharma S. R. Kapoor K. S. Gill H. S. (1995). Screening against sclerotinia rot (*Sclerotinia sclerotiarum*), downy mildew (*Peronospora parasitica*) and black rot (*Xanthomonas compestris*) in cauliflower (*Brassica oleracea* var. *botrytis* sub var. *cauliflora* DC). *Indian J. Agric. Sci.* 65 916–918.

[B165] Silue D. Nashaat N. I. Tirilly Y. (1996). Differential responses of *Brassica oleracea* and B. *rapa* accessions to seven isolates of *Peronospora parasitica* at the cotyledon stage. *Plant Dis.* 80 142–144.

[B166] Silue D. Laviec C. Tirilly Y. (1995). New sources of resistance in cauliflower (*Brassica oleracea var. botrytis*) to crucifer downy mildew caused by *Peronospora parasitica*. *J. Phytopathology* 143 659–661.

[B167] Simms E. L. (1992). “Costs of plant resistance to herbivory,” in *Plant Resistance to Herbivores and Pathogens: Ecology, Evolution, and Genetics*, eds Fritz R. S. Simms E. L. (Chicago: University of Chicago Press), 392–425.

[B168] Sinapidou E. Williams K. Nott L. Bahkt S. Tör M. Crute I. (2004). Two TIR: NB: LRR genes are required to specify resistance to *Peronospora parasitica* isolate Cala2 in *Arabidopsis*. *Plant J.* 38 898–909. 10.1111/j.1365-313X.2004.02099.x 15165183

[B169] Singh R. Trivedi B. M. Gill H. S. Sen B. (1987). Breeding for resistance to black rot, downy mildew and curd blight in Indian cauliflower. *Eucarpia Cruciferae Newsl.* 12 96–97.

[B170] Singh S. Sharma S. R. Kalia P. Deshmukh R. Katara J. Sharma P. (2015). Identification of putative DNA markers for disease resistance breeding in Indian cauliflower (*Brassica oleracea* var. *botrytis* L.). *Indian J. Biotechnol.* 14 455–460.

[B171] Singh S. Sharma S. R. Kalia P. Deshmukh R. Kumar V. Sharma P. (2012). Molecular mapping of the downy mildew resistance gene Ppa3 in cauliflower (*Brassica oleracea* var. *botrytis* L.). *J. Hortic. Sci. Biotechnol.* 87 137–143. 10.1080/14620316.2012.11512844

[B172] Singh S. Sharma S. R. Kalia P. Sharma P. Kumar V. Kumar R. (2013). Screening of cauliflower (*Brassica oleracea* var. *botrytis* L.) germplasm for resistance to downy mildew [*Hyaloperonospora parasitica* Constant (Pers.:Fr) Fr.] and designing appropriate multiple resistance breeding strategies. *Hortic. Sci. Biotechnol.* 88 103–109. 10.1080/14620316.2013.11512942

[B173] Singh S. Sidhu J. S. Huang N. Vikal Y. Li Z. Brar D. S. (2001). Pyramiding three bacterial blight resistance genes (xa5, xa13 and Xa21) using marker-assisted selection into indica rice cultivar PR106. *Theor. Appl. Genet.* 102 1011–1015.

[B174] Steiner B. Michel S. Maccaferri M. Lemmens M. Tuberosa R. Buerstmayr H. (2019). Exploring and exploiting the genetic variation of Fusarium head blight resistance for genomic-assisted breeding in the elite durum wheat gene pool. *Theor. Appl. Genet.* 132 969–988. 10.1007/s00122-018-3253-9 30506523 PMC6449325

[B175] Steuernagel B. Periyannan S. K. Hernández-Pinzón I. Witek K. Rouse M. N. Yu G. (2016). Rapid cloning of disease-resistance genes in plants using mutagenesis and sequence capture. *Nat. Biotechnol.* 34 652–655. 10.1038/nbt.3543 27111722

[B176] Stotz H. U. Mitrousia G. K. DeWit P. J. G. M. Fitt B. D. L. (2014). Effector triggered defence against apoplastic fungal pathogens. *Trends. Plant Sci.* 19 491–500. 10.1016/j.tplants.2014.04.009 24856287 PMC4123193

[B177] Sun D. Wang C. Zhang X. Zhang W. Jiang H. Yao X. (2019). Draft genome sequence of cauliflower (*Brassica oleracea* L. var. *botrytis*) provides new insights into the C genome in Brassica species. *Hortic. Res.* 6 82. 10.1038/s41438-019-0164-0 31645943 PMC6804732

[B178] Sun Q. Lin L. Liu D. Wu D. Fang Y. Wu J. (2018). CRISPR/Cas9-mediated multiplex genome editing of the *BnWRKY11* and *BnWRKY70* genes in *Brassica napus* L. *Int. J. Mol. Sci.* 19:2716. 10.3390/ijms19092716 30208656 PMC6163266

[B179] Sun Z. Li N. Huang G. Xu J. Pan Y. Wang Z. (2013). Site-Specific gene targeting using transcription activator-like effector (TALE)-based nuclease in *Brassica oleracea*. *J. Integr. Plant Biol.* 55 1092–1103. 10.1111/jipb.12091 23870552

[B180] Takagi H. Abe A. Yoshida K. Kosugi S. Natsume S. Mitsuoka C. (2013). QTL-seq: rapid mapping of quantitative trait loci in rice by whole genome resequencing of DNA from two bulked populations. *Plant J.* 74 174–183. 10.1111/tpj.12105 23289725

[B181] Thomas C. E. Jourdain E. L. (1990). Evaluation of broccoli and cauliflower germplasm for resistance to race 2 of *Peronospora parasitica*. *Hort. Sci.* 25 1429–1431.

[B182] Thomas C. E. Jourdain E. L. (1992). Resistance to race 2 of *peronospora parasitica* in U.S. plant introductions of *Brassica oleracea* var. *capitata*. *Hort Sci.* 27 1120–1122.

[B183] Tonguç M. Griffiths P. D. (2004). Genetic relationships of Brassica vegetables determined using database derived simple sequence repeats. *Euphytica* 137 193–201. 10.1023/B:EUPH.0000041577.84388.43

[B184] Tor M. Gordon P. Cuzick A. Eulgem T. Sinapidou E. Mert-Turk F. (2002). Arabidopsis SGT1b is required for defense signaling conferred by several downy mildew resistance genes. *Plant Cell* 14 993–1003. 12034892 10.1105/tpc.001123 PMC150602

[B185] Van der Biezen E. A. Freddie C. T. Kahn K. Parker J. E. Jones J. D. (2002). Arabidopsis RPP4 is a member of the RPP5 multigene family of TIR-NB-LRR genes and confers downy mildew resistance through multiple signalling components. *Plant J.* 29 439–451. 11846877 10.1046/j.0960-7412.2001.01229.x

[B186] Vera Cruz C. M. Bai J. Oña I. Leung H. Nelson R. J. Mew T. W. (2000). Predicting durability of a disease resistance gene based on an assessment of the fitness loss and epidemiological consequences of avirulence gene mutation. *Proc. Natl. Acad. Sci. U.S.A.* 97 13500–13505. 10.1073/pnas.250271997 11095723 PMC17604

[B187] Verma A. Singh Y. (2018). Inheritance of downy mildew resistance and its relationship with biochemical traits in cauliflower (*Brassica oleracea* L. var. *botrytis*). *Crop Prot.* 106 132–138.

[B188] Vicente J. G. Gunn N. D. Bailey L. Pink D. A. C. Holub E. B. (2012). Genetics of resistance to downy mildew in *Brassica oleracea* and breeding towards durable disease control for UK vegetable production. *Plant Pathol.* 61 600–609. 10.1111/j.1365-3059.2011.02539.X

[B189] Wang M. Farnham M. W. Thomas C. E. (2000). Phenotypic variation for downy mildew resistance among inbred broccoli. *Hort. Sci.* 35 925–929.

[B190] Wang T. Zhang H. Zhu H. (2019). CRISPR technology is revolutionizing the improvement of tomato and other fruit crops. *Hortic. Res.* 6:77. 10.1038/s41438-019-0159-x 31240102 PMC6570646

[B191] Wang X. Wang H. Wang J. Sun R. Wu J. Liu S. (2011). The genome of the mesopolyploid crop species*Brassica rapa*. *Nat. Genet.* 43 1035–1039. 10.1038/ng.919 21873998

[B192] Wang Y. Cheng X. Shan Q. Zhang Y. Liu J. Gao C. (2014). Simultaneous editing of three homoeoalleles in hexaploid bread wheat confers heritable resistance to powdery mildew. *Nat. Biotechnol.* 32 947–951. 10.1038/nbt.2969 25038773

[B193] Wang Y. VandenLangenberg K. Wen C. Wehner T. C. Weng Y. (2018). QTL mapping of downy and powdery mildew resistances in PI 197088 cucumber with genotyping-by-sequencing in RIL population. *Theor. Appl. Genet.* 131 597–611. 10.1007/s00122-017-3022-1 29159421

[B194] Win K. T. Vegas J. Zhang C. Song K. Lee S. (2017). QTL mapping for downy mildew resistance in cucumber via bulked segregant analysis using next-generation sequencing and conventional methods. *Theor. Appl. Genet.* 130 199–211. 10.1007/s00122-016-2806-z 27714417

[B195] Witek K. Jupe F. Witek A. I. Baker D. Clark M. D. Jones J. D. (2016). Accelerated cloning of a potato late blight-resistance gene using RenSeq and SMRT sequencing. *Nat. Biotechnol.* 34 656–660. 10.1038/nbt.3540 27111721

[B196] Wu L. Wang P. Wang Y. Cheng Q. Lu Q. Liu J. (2019). Genome-wide correlation of 36 agronomic traits in the 287 Pepper (*Capsicum*) accessions obtained from the SLAF-seq-based GWAS. *Int. J. Mol. Sci.* 20:5675. 10.3390/ijms20225675 31766117 PMC6888518

[B197] Xiao D. Liu S. T. Wei Y. P. Zhou D. Y. Hou X. L. Li Y. (2016). cDNA-AFLP analysis reveals differential gene expression in incompatible interaction between infected non-heading Chinese cabbage and *Hyaloperonospora parasitica*. *Hortic. Res.* 3:16034. 10.1038/hortres.2016.34 27602230 PMC4962739

[B198] Xu J. Hua K. Lang Z. (2019). Genome editing for horticultural crop improvement. *Hortic. Res.* 6:113. 10.1038/s41438-019-0196-5 31645967 PMC6804600

[B199] Xu L. Jiang Q. W. Wu J. Wang Y. Gong Y. Q. Wang X. L. (2014). Identification and molecular mapping of the RsDmR locus conferring resistance to downy mildew at seedling stage in radish (*Raphanus sativus* L.). *J. Integr. Agric.* 13 2362–2369. 10.1016/S2095-3119(14)60792-9

[B200] Yadav R. D. S. Singh S. B. Rai M. Singh S. N. Singh B. N. Maurya M. L. (1990). Gene pyramiding and horizontal resistance to diara stress in mustards. *Natl. Acad. Sci. Lett.* 13 325–327.

[B201] Yang H. Wu J. J. Tang T. Liu K. D. Dai C. (2017). CRISPR/Cas9-mediated genome editing efficiently creates specific mutations at multiple loci using one sgRNA in *Brassica napus*. *Sci. Rep.* 7:7489. 10.1038/s41598-017-07871-9 28790350 PMC5548805

[B202] Yang J. Liu D. Wang X. Ji C. Cheng F. Liu B. (2016). The genome sequence of allopolyploid *Brassica juncea* and analysis of differential homoeolog gene expression influencing selection. *Nat. Genet.* 48 1225–1232. 10.1038/ng.3657 27595476

[B203] Yang S. Li J. Zhang X. Zhang Q. Huang J. Chen J. Q. (2013). Rapidly evolving R genes in diverse grass species confer resistance to rice blast disease. *Proc. Natl. Acad. Sci. U.S.A.* 110 18572–18577. 10.1073/pnas.1318211110 24145399 PMC3831948

[B204] Yin K. Qiu J. L. (2019). Genome editing for plant disease resistance: applications and perspectives. *Philos. Trans. R. Soc. Lond. B. Biol. Sci.* 374:20180322. 10.1098/rstb.2018.0322 30967029 PMC6367152

[B205] Yoshioka Y. Sakata Y. Sugiyama M. Fukino N. (2014). Identification of quantitative trait loci for downy mildew resistance in cucumber (*Cucumis sativus* L.). *Euphytica* 198 265–276. 10.1007/s10681-014-1102-8

[B206] Yu J. Buckler E. S. (2006). Genetic association mapping and genome organization of maize. *Curr. Opin. Biotechnol.* 17 155–160. 10.1016/j.copbio.2006.02.003 16504497

[B207] Yu J. Tehrim S. Zhang F. Tong C. Huang J. Cheng X. (2014). Genomewide comparative analysis ofNBS-encoding genes between Brassica species and *Arabidopsis thaliana*. *BMC Genomics* 15:3. 10.1186/1471-2164-15-3 24383931 PMC4008172

[B208] Yu S. Su T. Zhi S. Zhang F. Wang W. Zhang D. (2016). Construction of a sequence-based bin map and mapping of QTLs for downy mildew resistance at four developmental stages in Chinese cabbage (*Brassica rapa* L. ssp. *pekinensis*). *Mol. Breed.* 36 1–12. 10.1007/s11032-016-0467-x

[B209] Yu S. C. Zhang F. L. Yu R. B. Zou Y. M. Qi J. N. Zhao X. Y. (2009). Genetic mapping and localization of a major QTL for seedling resistance to downy mildew in Chinese cabbage (*Brassica rapa* ssp. *pekinensis*). *Mol. Breed.* 23 573–590.

[B210] Yu S. C. Zhang F. L. Zhao X. Y. Yu Y. J. Zhang D. S. (2011). Sequence-characterized amplified region and simple sequence repeat markers for identifying the major quantitative trait locus responsible for seedling resistance to downy mildew in Chinese cabbage (*Brassica rapa* ssp. *pekinensis*). *Plant Breed.* 130 580–583.

[B211] Zeilmaker T. Ludwig N. R. Elberse J. Seidl M. F. Berke L. Van Doorn A. (2015). DOWNY mildew resistant 6 and DMR6-like OXYGENASE 1 are partially redundant but distinct suppressors of immunity in *Arabidopsis*. *Plant J.* 81 210–222. 10.1111/tpj.12719 25376907

[B212] Zhai Y. Cai S. Hu L. Yang Y. Amoo O. Fan C. (2019). CRISPR/Cas9-mediated genome editing reveals differences in the contribution of INDEHISCENT homologues to pod shatter resistance in *Brassica napus* L. *Theor. Appl. Genet.* 132 2111–2123. 10.1007/s00122-019-03341-0 30980103

[B213] Zhai Y. Yu K. Cai S. Hu L. Amoo O. Xu L. (2020). Targeted mutagenesis of BnTT8 homologs controls yellow seed coat development for effective oil production in *Brassica napus* L. *Plant Biotechnol. J.* 18 1153–1168. 10.1111/pbi.13281 31637846 PMC7152602

[B214] Zhang B. Li P. Su T. Li P. Xin X. Wang W. (2018). BrRLP48, encoding a receptor-like protein, involved in downy mildew resistance in *Brassica rapa*. *Front. Plant Sci.* 9:1708. 10.3389/fpls.2018.01708 30532761 PMC6265505

[B215] Zhang B. Su T. Li P. Xin X. Cao Y. Wang W. (2021). Identification of long noncoding RNAs involved in resistance to downy mildew in Chinese cabbage. *Hortic. Res.* 8:44. 10.1038/s41438-021-00479-1 33642586 PMC7917106

[B216] Zhang H. Zhang J. Wei P. Zhang B. Gou F. Feng Z. (2014). The CRISPR/Cas9 system produces specific and homozygous targeted gene editing in rice in one generation. *Plant Biotechnol. J.* 12 797–807. 10.1111/pbi.12200 24854982

[B217] Zhang K. Nie L. Cheng Q. Yin Y. Chen K. Qi F. (2019). Effective editing for lysophosphatidic acid acyltransferase 2/5 in allotetraploid rapeseed (*Brassica napus* L.) using CRISPR-Cas9 system. *Biotechnol. Biofuels* 12:225. 10.1186/s13068-019-1567-8 31548867 PMC6753616

[B218] Zhang L. Zhou Q. (2014). CRISPR/Cas technology: a revolutionary approach for genome engineering. *Sci China Life Sci.* 57 639–640. 10.1007/s11427-014-4670-x 24849511

[B219] Zhang L. Wang M. Li N. Wang H. Qiu P. Pei L. (2018). Long noncoding RNAs involve in resistance to *Verticillium dahliae*, a fungal disease in cotton. *Plant Biotechnol. J.* 16 1172–1185. 10.1111/pbi.12861 29149461 PMC5978870

[B220] Zhang S. J. Yu S. C. Zhang F. L. Zhao X. Y. Yu Y. J. Zhang D. S. (2012). Inheritance of downy mildew resistance at different developmental stages in Chinese cabbage via the leaf disk test. *Hort. Environ. Biotechnol* 3 397–403.

[B221] Zhang S. P. Liu M. M. Miao H. Zhang S. Q. Yang Y. H. Xie B. Y. (2013). Chromosomal mapping and QTL analysis of resistance to downy mildew in *Cucumis sativus*. *Plant Dis.* 97 245–251. 10.1094/PDIS-11-11-0941-RE 30722314

[B222] Zhang Z. Ge X. Luo X. Wang P. Fan Q. Hu G. (2018). Simultaneous editing of two copies of Gh14-3-3d confers enhanced transgene-clean plant defense against *Verticillium dahliae* in allotetraploid upland cotton. *Front. Plant Sci.* 9:842. 10.3389/fpls.2018.00842 30013582 PMC6036271

[B223] Zhao Z. Gu H. Sheng X. Yu H. Wang J. Zhao J. (2015). Genetic diversity and relationships among loose-curd cauliflower and related varieties as revealed by microsatellite markers. *Sci. Hortic.* 166 105–110.

[B224] Zheng H. Zhang Y. Li J. He L. Wang F. Bi Y. (2020). Comparative transcriptome analysis between a resistant and a susceptible Chinese cabbage in response to *Hyaloperonospora brassicae*. *Plant Signal Behav.* 15:1777373. 10.1080/15592324.2020.1777373 32538253 PMC8570763

[B225] Zheng M. Zhang L. Tang M. Liu J. Liu H. Yang H. (2020). Knockout of two BnaMAX1 homologs by CRISPR/Cas9-targeted mutagenesis improves plant architecture and ncreases yield in rapeseed (*Brassica napus* L.). *Plant Biotechnol. J.* 18 644–654. 10.1111/pbi.13228 31373135 PMC7004912

[B226] Zhi S. Su T. Yu S. Zhang F. Yu Y. Zhang D. (2016). Genetic characteristics of A01-located resistant loci to downy mildew in Chinese cabbage by genome-wide association studies. *Plant Physiol. J.* 52 693–702. 10.13592/j.cnki.ppj.2016.0026

[B227] Zhong X. Zhou Q. Cui N. Cai D. Tang G. (2019). BvcZR3 and BvHs1(pro-1) genes pyramiding enhanced beet cyst nematode (*Heterodera schachtii Schm*.) resistance in oilseed rape (*Brassica napus* L.). *Int. J. Mol. Sci.* 20:1740. 10.3390/ijms20071740 30965683 PMC6479909

[B228] Zhou J. M. Tang X. Y. Martin G. B. (1997). The Pto kinase conferring resistance to tomato bacterial speck disease interacts with proteins that bind a cis-element of pathogenesis-related genes. *EMBO J.* 16 3207–3218. 10.1093/emboj/16.11.3207 9214637 PMC1169938

[B229] Zhu S. Zhang X. Liu Q. Luo T. Tang Z. Zhou Y. (2018). The genetic diversity and relationships of cauliflower (*Brassica oleracea* var. *botrytis*) inbred lines assessed by using SSR markers. *PLoS One* 13:e0208551. 10.1371/journal.pone.0208551 30521622 PMC6283626

[B230] Zuo W. Chao Q. Zhang N. Ye J. Tan G. Li B. (2015). A maize wall associated kinase confers quantitative resistance to head smut. *Nat. Genet.* 47 151–157. 10.1038/ng.3170 25531751

